# Predicting per- and polyfluoroalkyl substances removal in pilot-scale granular activated carbon adsorbers from rapid small-scale column tests

**DOI:** 10.1002/aws2.1369

**Published:** 2024-03-19

**Authors:** Zachary R. Hopkins, Detlef R. U. Knappe

**Affiliations:** Department of Civil, Construction, and Environmental Engineering, North, Carolina State University, Raleigh, North, Carolina, USA

**Keywords:** hexafluoropropylene oxide-dimer acid (HFPO-DA, GenX), perfluorooctane sulfonate (PFOS), perfluorooctanoic acid (PFOA), remediation

## Abstract

Per- and polyfluoroalkyl substances (PFAS) occur widely in drinking water, and consumption of contaminated drinking water is an important human exposure route. Granular activated carbon (GAC) adsorption can effectively remove PFAS from water. To support the design of GAC treatment systems, a rapid bench-scale testing procedure and scale-up approach are needed to assess the effects of GAC type, background water matrix, and empty bed contact time (EBCT) on GAC use rates. The overarching goal of this study was to predict PFAS breakthrough curves obtained at the pilot-scale from rapid small-scale column test (RSSCT) data. The scale-up protocol was developed for pilot data obtained with coagulated/settled surface water (TOC = 2.3 mg/L), three GACs, and two EBCTs. Between 7 and 11 PFAS breakthrough curves were available for each pilot column. RSSCT designs were investigated that assumed intraparticle diffusivity is independent of GAC particle size (i.e., constant diffusivity [CD]) or linearly dependent on GAC particle size (i.e., proportional diffusivity [PD]). CD-RSSCTs effectively predicted the bed volumes of water that could be treated at the pilot-scale to reach 50% breakthrough (${\text{BV}}_{50\%}$) of individual PFAS. In contrast, PD-RSSCTs overpredicted ${\text{BV}}_{50\%}$ obtained at the pilot-scale by a factor of ~2–3. The shape of PFAS breakthrough curves obtained with CD-RSSCTs deviated from those obtained at the pilot-scale, indicating that intraparticle diffusivity was dependent on GAC particle diameter $\left(d_{\text{p}}\right)$. Using the pore surface diffusion model (PSDM), intraparticle diffusivity was found to be proportional to $\left(d_{\text{p}}\right)$^0.25^ when considering data up to about 70% PFAS breakthrough. This proportionality factor can be used to design RSSCTs or scale up existing CD-RSSCT data using the PSDM. Using pilot-scale data obtained with groundwater and wastewater-impacted groundwater as well as with additional GACs, the developed RSSCT scale-up approach was validated for PFAS breakthrough percentages up to 70%. The presented methodology permits the rapid prediction of GAC use rates for PFAS removal.

## 1 | INTRODUCTION

Per- and polyfluoroalkyl substances (PFAS) are a broad class of xenobiotic chemicals that are receiving extensive public and research attention because of their ubiquity in human blood serum and the environment (Cousins et al., 2019; Evich et al., 2022; Glassmeyer et al., 2017; Hu et al., 2016). PFAS are extensively used in consumer and industrial products, and PFAS releases into the environment can occur throughout the entire life cycle of PFAS-containing products. Example products containing PFAS include stain repellents, non-stick cookware, lubricants, food packaging, cosmetics, and fire-fighting foams (Glüge et al., 2020; OECD, 2018; Wang et al., 2017). Vectors by which PFAS can enter the aquatic environment include use of aqueous film-forming foams (AFFF) in firefighting and firefighting training, treated municipal and industrial wastewater, biosolids generated during wastewater treatment, leachate from solid waste landfills, and air emissions from industrial facilities (Brendel et al., 2018; Guelfo et al., 2018; Hopkins et al., 2018; Hu et al., 2016; Sun et al., 2016; Washington et al., 2020). The persistence and mobility of PFAS has resulted in widespread global contamination of groundwater and surface water bodies that serve as a source of drinking water (Pan et al., 2018; Washington et al., 2020).

The detection of PFAS in drinking water has sparked public concern. Epidemiological studies suggest there is a link between exposure to PFAS and various adverse health effects including reduced immune response to vaccines, high cholesterol, ulcerative colitis, thyroid disease, testicular cancer, kidney cancer, breast cancer, and pregnancy induced hypertension (Foguth et al., 2020; Grandjean et al., 2012; Hurley et al., 2018; Kotlarz et al., 2020; NASEM, 2022). The bioaccumulative and toxic properties of long-chain PFAS (i.e., perfluoroalkylcarboxylic acids [PFCAs] with seven or more fully fluorinated methyl/methylene groups, pefluoroalkylsulfonic acids [PFSAs] with six or more fully fluorinated methyl/methylene groups) have led to the phase-out of their production and use in the United States and Europe. In their place, a shift toward shorter-chain PFAS and fluorinated alternatives, such as per- and polyfluoroalkyl ether acids (PFEAs), has taken place since about 2000 (Ritscher et al., 2018; Wang et al., 2017). To reduce exposure to PFAS, both federal and state-level agencies established non-enforceable health advisory levels (HALs) and enforceable maximum contaminant levels (MCLs) for PFAS in drinking water (ITRC, 2024; USEPA, 2022). HALs and MCLs are becoming increasingly more stringent in the United States and around the globe. For example, the United States Environmental Protection Agency’s (USEPA’s) draft lifetime HALs for perfluorooctanoic acid (PFOA) and perfluorooctanesulfonic acid (PFOS) are 0.004 and 0.02 ng/L, respectively, while final lifetime HALs for hexafluoropropylene oxide dimer acid (GenX) and perfluorobutanesulfonic acid (PFBS) are 10 and 2000 ng/L, respectively (USEPA, 2022). In addition, the USEPA proposed enforceable drinking water standards for PFOA and PFOS at 4 ng/L each as well as a hazard index approach for the sum of PFBS, PFHxS, perfluorononanoic acid (PFNA), and GenX (USEPA, 2023a). In Denmark, the MCL for the summed concentration of PFOA, PFNA, PFHxS, and PFOS is 2 ng/L (ITRC, 2024). As a result, drinking water purveyors need to implement treatment strategies that (cost-)effectively remove PFAS from their source water.

Many conventional and advanced treatment processes (e.g., coagulation, flocculation, sedimentation, filtration, oxidation processes) are ineffective for the removal or destruction of PFAS (Boone et al., 2019; Gebbink et al., 2017; Hopkins et al., 2018; Rahman et al., 2014). Granular activated carbon (GAC) adsorption, ion exchange, and high-pressure membrane filtration are readily implementable treatment approaches that can remove PFAS from drinking water (Appleman et al., 2014; Hopkins et al., 2018; Sun et al., 2016; Zhi & Liu, 2015). Among them, GAC adsorption is most widely implemented. PFAS removal by GAC has been evaluated at the bench-, pilot-, and full-scale (CFPUA, 2018, personal communication; Dickenson & Higgins, 2016; Eschauzier et al., 2012; Hopkins et al., 2018; Park et al., 2020; Rodowa et al., 2020; Zeng et al., 2020). Conducting pilot-scale adsorption studies is time-intensive and requires large volumes of water. The rapid small-scale column test (RSSCT) was developed to reduce the time and cost required for determining GAC adsorber performance (Crittenden et al., 1986, 1987). However, there is no validated protocol to scale up PFAS breakthrough curves obtained with RSSCTs.

The design of RSSCTs is based on the principle of similitude using parameters derived from the dimensionless form of the pore surface diffusion model. To reduce time and water requirements for the acquisition of micropollutant breakthrough curves, GAC used in RSSCTs is crushed to a smaller particle size. The ratio of the mean GAC particle diameter used in full- or pilot-scale studies ($d_{\text{LC}}$) to that used in RSSCTs ($d_{\text{SC}}$) is called the scaling factor $\left(\text{SF}=d_{\text{LC}}/d_{\text{SC}}\right)$. Two common RSSCT designs used to assess micropollutant removal from water are the constant diffusivity design (CD-RSSCT) and the proportional diffusivity design (PD-RSSCT). The CD-RSSCT design assumes intraparticle diffusivity is independent of particle size while the PD-RSSCT design assumes intraparticle diffusivity increases linearly with particle size. The CD-RSSCT design has been used to determine how PFAS and sorbent characteristics affect PFAS removal effectiveness (Zeng et al., 2020), but limited information is available on scale-up of CD-RSSCT data (Park et al., 2020; Schaefer et al., 2020). Park et al. (2020) conducted CD-RSSCTs using different GAC particle sizes and found that intraparticle diffusion coefficients were independent of GAC particle size over a limited size range (0.13–0.2 mm). Schaefer et al. (2020) suggested that CD-RSSCTs effectively simulated PFAS adsorption kinetics obtained with as-received GAC, but RSSCTs overpredicted PFAS adsorption capacities of GAC. In Schaefer et al. (2020), the SF was only ~3.5, and column studies conducted with as-received sorbents did not simulate hydraulic conditions typically used in full-scale column processes. Ideally, larger SF values (smaller GAC diameters) would be used in RSSCTs to shorten RSSCT duration and minimize water requirements.

For neutral organic micropollutants, PD-RSSCT data have been used as a basis for developing scale-up approaches because PD-RSSCT data effectively described the adsorption kinetics of neutral organic micropollutants (Corwin & Summers, 2010; Kennedy et al., 2017). However, PD-RSSCT data overpredict the full-scale adsorption capacity of GAC for organic micropollutants by a factor of about 3, necessitating the use of a fouling index to predict full-scale GAC performance from PD-RSSCT data (Corwin & Summers, 2010). While the fouling index approach can be used effectively to scale up PD-RSSCT data, the collection of PD-RSSCT data is time-intensive (weeks to months) and requires relatively large volumes of water (hundreds of liters). In the context of PFAS removal, Kempisty et al. (2022) conducted PD-RSSCTs and found that resulting PFAS adsorption capacities overpredicted full-scale performance by a factor of 2. Thus, the fouling index that applies to anionic perfluoroalkyl acids may be smaller than for neutral organic micropollutants, but PD-RSSCTs nonetheless overpredicted adsorption capacities.

The primary objective of this research was to develop a scale-up approach to predict pilot-scale GAC performance for PFAS removal from RSSCT data. A goal for the experimental portion of the scale-up procedure was to complete RSSCTs rapidly and with small volumes of water. To develop and validate the scale-up procedure, RSSCT data were matched with pilot-scale GAC adsorber data that investigated the removal of 11 PFAS by six GACs from coagulated surface water, groundwater, and wastewater-impacted groundwater.

## 2 | MATERIALS AND METHODS

### 2.1 | Materials

#### 2.1.1 | Per- and polyfluoroalkyl substances

RSSCTs were conducted with 22 PFAS spiked into RSSCT influent water, including seven PFCAs, three PFSAs, one fluorotelomer sulfonic acid (FtS), nine perfluoroalkylether carboxylic acids (PFECAs), and one polyfluoroalkyl ether sulfonic acid (PFESA) as summarized in Table S1. PFAS were obtained from Synquest Laboratories Inc. (Alachua, FL) and The Chemours Company (Wilmington, DE). In addition, native and mass-labeled PFAS standards used for PFAS analysis by liquid chromatography-tandem mass spectrometry were obtained from Wellington Laboratories (Guelph, ON).

#### 2.1.2 | Water

RSSCTs and pilot-scale tests were conducted in matching water matrices obtained from two drinking water utilities: Water A (TOC = 2.3 mg/L) was PFAS-impacted surface water from a utility in USEPA Region 4 that had undergone raw water ozonation, coagulation, flocculation, sedimentation, settled water ozonation, and biofiltration (subsequently called coagulated surface water), and Water B (TOC < 0.5 mg/L) was PFAS-impacted groundwater from a utility in USEPA Region 2 that did not receive any pretreatment. In addition, matching RSSCT and pilot column data were analyzed that had been obtained with a wastewater-impacted groundwater (Water C) from a utility in USEPA Region 9 (Medina et al., 2022). PFAS concentrations and background water quality parameters (TOC concentration, UV_254_ absorbance) for the influents of RSSCTs and pilot-scale adsorbers are provided in Tables S1 and S2, respectively. For RSSCTs, a batch of Water A was collected that matched the average TOC concentration of the influent to the pilot columns (2.3 mg/L). TOC concentrations in pilot column and RSSCT influents were also similar for Water B (<0.5 mg/L) and C (1.3 and 1.6 mg/L, Table S2; Medina et al., 2022). For preliminary RSSCTs, in which the effect of GAC particle size used in RSSCTs was evaluated and CD- and PD-RSSCT designs were compared, additional water samples were obtained from Utility A, which had TOC concentrations of 1.3 and 1.5 mg/L (Table S2). For RSSCTs, Water A and Water B were stored in high density polypropylene containers (200-L drums, 20-L carboys) at 4C until use. Prior to use in RSSCTs, water was filtered through a 5-μm polypropylene cartridge filter (model #P5, Culligan).

For RSSCTs conducted with Water A and B, 22 PFAS were spiked at concentrations ranging from 85 to 158 ng/L (Table S1). In RSSCTs, PFAS were dosed into the influent water after cartridge filtration to (1) achieve concentrations that permitted measurement of the onset of PFAS breakthrough at an effluent concentration corresponding to <5% of the influent concentration and (2) to obtain breakthrough curves for PFAS that were present at low or nonquantifiable levels in the collected water. All pilot studies were conducted with PFAS that were present in the source water (Table S1). While the influent concentrations between RSSCTs and pilot columns differed, ongoing RSSCT studies in the authors’ laboratory show that percent PFAS breakthrough as a function of bed volumes of water treated is independent of the influent PFAS concentration over a 50-fold concentration range (from 0.2 to 10 nM, Meng et al., 2022). Pilot-scale and RSSCT data for PFOA removal from wastewater-impacted groundwater (Water C from a utility in US EPA Region 9) were provided by Jacobs Engineering Group Inc. and were used for validation of the developed scale-up protocol (Medina et al., 2022).

#### 2.1.3 | Granular activated carbon

For Water A, RSSCTs and pilot tests were conducted with two bituminous coal-based carbons (GAC 1 and GAC 2) and one enhanced coconut shell-based carbon (GAC 3). For Water B, RSSCTs and pilot tests were conducted with GAC 1. For Water C, RSSCTs and pilot tests were conducted with three bituminous coal-based carbons (GAC 1, GAC 4, and GAC 6) and one lignite-based carbon (GAC 5). Information about GAC properties is provided in Table S3. GACs were used in as-received form for pilot-scale testing. For RSSCTs, GAC was wetted with ultrapure water (UPW), crushed with a mortar and pestle, wet-sieved, and the fractions in the size ranges of 63–74 μm (200 × 230 U.S. Standard Mesh), 74–149 μm (100 × 200 U.S. Standard Mesh), and 44–63 μm (230 × 325 U.S. Standard Mesh) were collected. The 100 × 200 US Standard Mesh size was selected to be consistent with previous RSSCT research (Kempisty et al., 2022; Summers et al., 2014). Prior to use, GAC was dried at 105C and stored in a desiccator.

### 2.2 | Methods

#### 2.2.1 | Rapid small-scale column tests

RSSCTs were based on two common designs, the CD-RSSCT and the PD-RSSCT designs (Equation 1, Text S1; Crittenden et al., 1986, 1987; Hand et al., 1987; Summers et al., 2014).

(1) $$\left[\frac{{\text{EBCT}}_{\text{RSSCT }}}{{\text{ EBCT }}_{\text{pilot-scale }}}\right]={\left[\frac{d_{\text{pilot-scale }}}{d_{\text{RSSCT }}}\right]}^{X-2}={\left(\text{SF}\right)}^{X-2},$$

where EBCT is the empty bed contact time, d is the GAC diameter (the log-mean diameter was used in this study to be consistent with earlier studies; Kempisty et al., 2022; Summers et al., 2014), SF is the scaling factor, and X is the proportionality factor, which takes on values of 0 and 1 for CD- and PD-RSSCTs, respectively. For Water A and Water B, scaling factors for the CD- and PD-RSSCT designs ranged from 13.3 to 15.4 depending on the GAC (Tables S4 and S6). For Water C, CD-RSSCT data were obtained with SF values of 7.2–7.7 (Table S7).

To reduce the required water volume and minimize the risk of column clogging, CD-RSSCTs were operated with a lower flow rate than what is required for perfect similitude. The hydraulic loading rate (HLR) was selected such that the product of the Reynolds and Schmidt numbers (Re × Sc) was >400 for PFMOAA, the smallest evaluated PFAS—a minimum Re × Sc value of 200 is required for the mechanical dispersion region, in which dispersion phenomena are not overemphasized (Text S1, Berrigan, 1985). The Reynolds number was calculated from the log-mean particle diameter. Applying the concept of a minimum Reynolds number to CD-RSSCTs is analogous to what Crittenden et al. (1987) proposed for PD-RSSCTs (Equation 2, Crittenden et al., 1987).


(2) HLRRSSCT =HLRpilot-scale (dpilot-scale dRSSCT)(ReRSSCT,min Repilot-scale ).

To assess whether the reduced HLR for CD-RSSCTs is reasonable, PFAS breakthrough curves were compared for CD-RSSCT designs with ideal and reduced HLRs using the pore surface diffusion model (PSDM). Simulated breakthrough curves were almost identical for the two designs, suggesting that reduced HLRs, such as the ones selected here (~10.7 m/h), can be used to simulate PFAS adsorption in CD-RSSCTs (Figure S1).

CD-RSSCTs were initially conducted using stainless steel guard columns (inside diameter [ID] = 0.4 cm, length [L] = 1 cm, Table S4). Because these columns are relatively expensive and do not permit visual observation of the GAC bed during testing, RSSCTs were redesigned using polypropylene (PP) tubing with ID = 3.18 mm to hold the GAC bed for CD-RSSCTs (Tables S4–S6). Sorbent-free control experiments (PFAS-free, PFAS-spiked) showed that the experimental apparatus neither leached nor sorbed the PFAS targeted in this study (Cheng & Knappe, 2024). Despite the small particle sizes and short GAC bed depths used for CD-RSSCTs, GAC beds could be prepared in a reproducible manner, and no operational problems, such as pressure buildup, were encountered. Details about the preparation of RSSCT columns are described in Text S1. PD-RSSCTs were designed in a manner similar to Kennedy et al. (2017), using PP tubing with ID = 4.76 mm (Tables S4 and S5). PFAS-spiked water was stored in a 55-gal drum or 5-gal carboy, depending on water requirements, and pumped through the columns with a HPLC pump (Shimadzu AT-20, Columbia, MD). Two sampling points were installed to collect water samples before and after the GAC bed. Samples were collected in 15- or 50-mL polypropylene centrifuge tubes (Fisher, Waltham, MA) for PFAS analysis and 40-mL amber glass vials (ESS, San Leandro, CA) for TOC/UV254 analysis. Influent samples were collected at the start and end of each RSSCT. For CD-RSSCTs, effluent samples were collected every 500–1000 bed volumes (BV) for the first 10,000 BV and every 5000–10,000 BV for the remainder of the experiment. For PD-RSSCTs, effluent samples were collected every 2500 BV for the first 10,000 BV and every 5000–10,000 BV thereafter. RSSCTs conducted with Water A (Table S2) were operated for ~86,000–120,000 BV and ~70,000–85,000 BV for the CD and PD designs, respectively. For Water B, CD-RSSCTs were operated for 98,000 BV. Samples for PFAS and TOC/UV254 analysis were stored at 4C prior to analysis.

To validate the scale-up method developed with Water A, breakthrough curves for perfluorobutanoic acid (PFBA), perfluoropentanoic acid (PFPeA), and perfluorohexanoic acid (PFHxA) from a pilot study and a CD-RSSCT conducted with Water B (Tables S1 and S2) were compared. Additionally, CD-RSSCT data for a wastewater-impacted groundwater were obtained from Jacobs Engineering (Water C, Tables S1, S2, and S7, Medina et al., 2022). Only PFOA breakthrough data were received for this data set, which was obtained with GACs 1, 4, 5, and 6.

#### 2.2.2 | Pilot-scale studies

The scale-up approach was developed with data from pilot studies conducted with Water A and validated with data from pilot studies conducted with Water B and C. Design specifications for the pilot studies conducted with Water A and B are summarized in Tables S4 and S6 while those for Water C are summarized in Medina et al. (2022). Pilot-scale adsorbers were operated to approximately 22,000 and 80,000 BV for Water A and B, respectively. Pilot-scale adsorbers operated with Water C were operated to approximately 70,000 BV (Medina et al., 2022).

#### 2.2.3 | PFAS analysis

RSSCT samples obtained with Water A and B were analyzed for PFAS by liquid chromatography-tandem mass spectrometry (LC-MS/MS, 1260 HPLC, Ultivo MS, Agilent Technologies, Inc., Santa Clara, CA) at North Carolina State University. A full description of the analytical method is provided in Text S2. Briefly, a large-volume injection method was used to determine PFAS concentrations without prior solid-phase extraction. An isotope dilution method was used to determine PFAS concentrations for compounds, for which isotopically labeled analogs were available. For other PFAS, internal standards with similar retention times and structures were used (Text S2). Calibration standards were prepared in UPW at concentrations ranging from 1 to 500 ng/L for all targeted PFAS. Accredited laboratories were used for determining PFAS concentrations in samples collected from all pilot-scale tests and from RSSCTs conducted with Water C (Medina et al., 2022).

#### 2.2.4 | Mathematical modeling

To determine the dependence of intraparticle diffusion on GAC particle size, pilot-scale and RSSCT data were described with the pore surface diffusion model (PSDM, AdDesignS, USEPA, 2023b). The PSDM is frequently used to describe micropollutant breakthrough curves (Crittenden et al., 1986; Kennedy et al., 2017; Rodowa et al., 2020). Input parameters include both system design and operating parameters (e.g., GAC particle diameter, bed density, bed diameter, bed depth, GAC mass, volumetric water flow rate) and physical/chemical adsorbate properties. In addition, adsorption equilibrium (i.e., Freundlich K and 1/n values) and kinetic parameters (film mass transfer coeffiecient [$k_{f}$], tortuosity [τ], surface-to-pore diffusion flux ratio [SPDFR]) are required. To obtain equilibrium and kinetic parameters, the pseudo-single-solute approach described in Rodowa et al. (2020) was used. Briefly, the Freundlich adsorption capacity parameter K was first estimated from ${\text{BV}}_{50\text{s}}/\rho_{\text{bed }}$, where ${\text{BV}}_{50\%}$ represents the bed volumes of water that can be treated to 50% breakthrough of a given PFAS, and ${\text{ρ}}_{\text{bed}}$ is the GAC bed density (Corwin & Summers, 2011). The Freundlich exponent 1/n was kept at a value of 1, which is consistent with the observation that normalized PFAS breakthrough curves are independent of the PFAS concentration in the GAC influent for concentrations up to at least 10 nM, which, for PFOS, corresponds to a mass concentration of ~5000 ng/L (Meng et al., 2022). To determine the kinetic parameters τ and SPDFR, τ was initially set to 1 and SPDFR to 10^−30^. If the initial model output resulted in a breakthrough curve that was steeper than indicated by the experimental data, τ was increased until a good match was obtained between the PSDM output and the experimental data. If the initial model output resulted in a breakthrough curve that was flatter than the experimental data, SPDFR was increased until model results matched the experimental data well. Good agreement between experimental data and model outputs was typically obtained from the onset of breakthrough until ~60%–70% PFAS breakthrough for all PFAS. In some cases, experimental data and model outputs diverged at higher breakthrough percentages, most likely because a pseudo-single-solute modeling approach was applied; in other words, GAC fouling by dissolved organic matter (DOM) and adsorption competition were not explicitly modeled because their inclusion would have increased the number of adjustable model parameters. Instead, the effect of DOM on PFAS adsorption was captured by system-specific equilibrium and kinetic parameters.

## 3 | RESULTS AND DISCUSSION

### 3.1 | Effect of granular activated carbon particle size on PFAS breakthrough curves

RSSCTs were conducted to determine whether the GAC particle size used in CD- and PD-RSSCTs affects PFAS breakthrough curves. CD-RSSCTs were conducted with coagulated surface water with a TOC of 1.5 mg/L and PD-RSSCTs with coagulated surface water from the same source, but with a TOC of 1.3 mg/L. Water for RSSCTs was collected from the same utility at different times; therefore, the initial TOC of the water for the PD-RSSCT and CD-RSSCT differed slightly. For CD-RSSCTs, log-mean particle diameters of 0.069 mm (200 × 230 US mesh) and 0.053 mm (230 × 325 US mesh) were compared while log-mean particle diameters of 0.069 mm (200 × 230 US mesh) and 0.11 mm (100 × 200 US mesh) were compared for PD-RSSCTs. Complete design specifications for the RSSCTs are provided in Table S5. Bed volumes that could be treated to 10%, 20%, 50% breakthrough for the 22 spiked PFAS are compared in parity plots for 22 PFAS (Figure S2). Results in Figure S2 demonstrate that the bed volumes of water that could be treated to 10%, 20%, and 50% breakthrough were similar for the evaluated particle diameters in the CD- and PD-RSSCTs. Additionally, describing the data with the PSDM yielded similar Freundlich adsorption capacity and adsorption kinetic parameters (Table S8). For crushed GAC, results presented in Figure S2 therefore suggest that intraparticle diffusivity was independent of particle size for both CD- and PD-RSSCTs under the tested condition. Park et al. (2020) demonstrated a similar independence of intraparticle diffusivity on crushed GAC particle size for CD-RSSCTs conducted with mean GAC particle diameters of 0.13, 0.17, and 0.2 mm. These results suggest RSSCT data obtained with GAC particle diameters commonly used in RSSCTs should be sufficiently similar that a common scale-up approach can be used. Although results of this study as well as those of others (Park et al., 2020; Schaefer et al., 2020) suggest intraparticle diffusion of PFAS is independent of GAC particle size when the range of GAC particle sizes is relatively narrow, information is lacking about the dependence of intraparticle diffusivity on GAC particle size for larger scaling factors, such as those typically encountered when linking RSSCT data to full- or pilot-scale data.

### 3.2 | Direct comparison of RSSCTs and pilot-scale adsorbers

**FIGURE 1:**
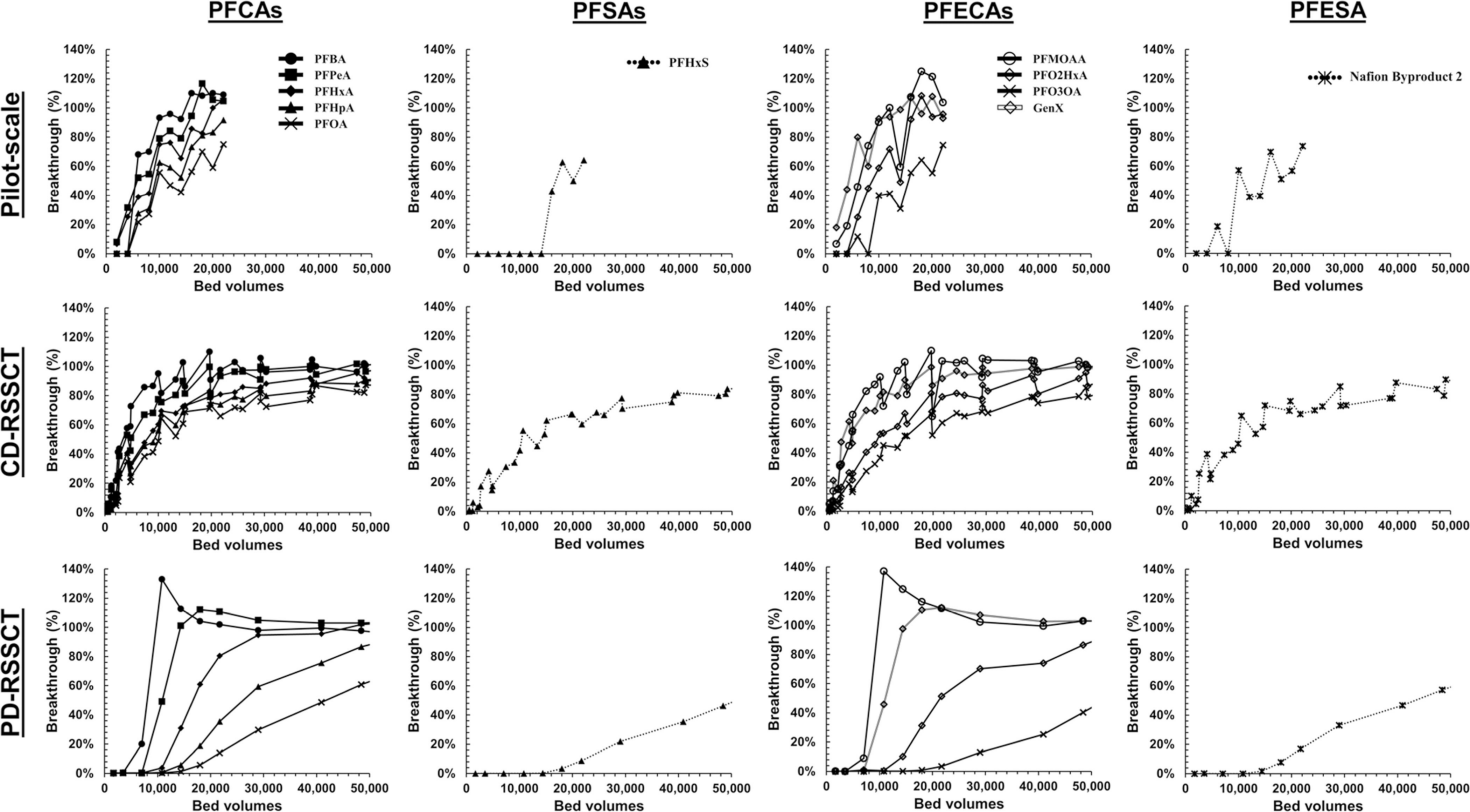
Per- and polyfluoroalkyl substances breakthrough curves obtained in Water A (TOC = 2.3 mgL^−1^) in a pilot-scale adsorber, constant diffusivity-rapid small-scale column test, and proportional diffusivity-rapid small-scale column test. Carbon: granular activated carbon 1, simulated empty bed contact time: 10 min.

To assess whether PFAS breakthrough curves obtained at the pilot-scale can be directly predicted from CD- or PD-RSSCT data, breakthrough curves were compared for the following scenarios: Water A with three GACs at an EBCT of ~10 min and with one GAC at an EBCT of ~20 min. As shown in Figure 1 for GAC 1 at an EBCT of ~10 min, directly comparable breakthrough curves for the RSSCT and pilot were available for 5 PFCAs, 1 PFSA, 4 PFECAs, and 1 PFESA. Similarly, 10 PFAS breakthrough curves were available for GAC 2 (Figure S3) and seven for GAC 3 (Figure S4) at an EBCT of ~10 min. At an EBCT of ~20 min, eight directly comparable PFAS breakthrough curves were available for GAC 1 (Figure S5). As is well known from other studies (Hopkins et al., 2018; Park et al., 2020; Rodowa et al., 2020), these data sets illustrate that PFAS adsorbability increases with increasing PFAS chain length within a given subclass (e.g., PFBA < PFPeA < PFHxA < PFHpA < PFOA for PFCAs and PFMOAA < PFO2HxA < PFO3OA for PFECAs). These trends were observed for both pilot-scale and RSSCT data (Figure 1).

**FIGURE 2:**
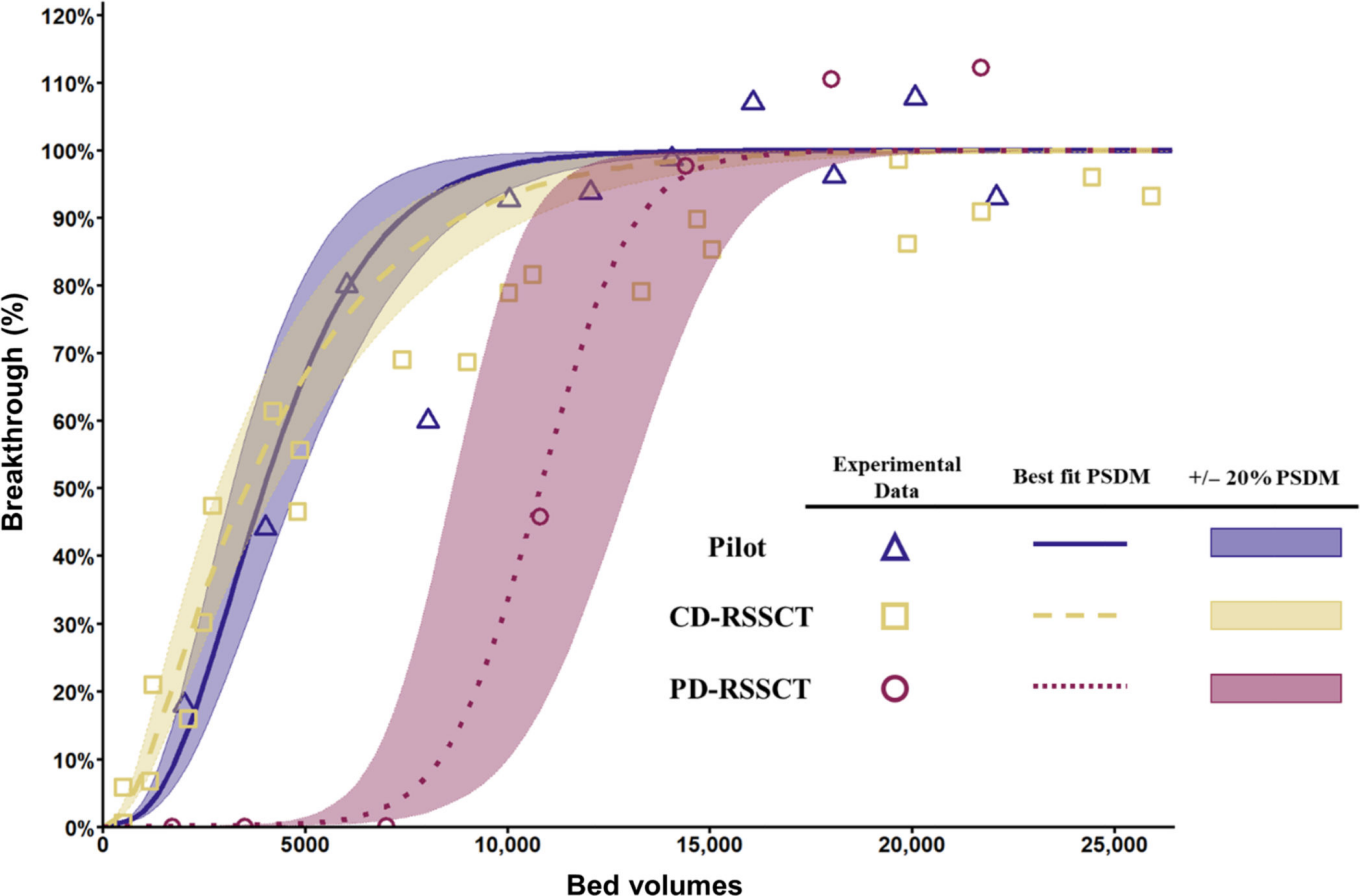
Direct comparison of GenX breakthrough curves obtained at the pilot-scale and in constant diffusivity- and proportional diffusivity-rapid small-scale column tests. Water A (TOC = 2.3 mg/L), Carbon: granular activated carbon 1, pilot-scale empty bed contact time: 10 min. Symbols represent experimental data and lines represent best fit of the pore surface diffusion model (PSDM) for each data set; the shaded region represents 20% variance around the best PSDM fit.

To formally compare pilot-scale and RSSCT results, all raw data were described with the PSDM, and resulting PFAS breakthrough curves were plotted as shown in Figure 2 for the GenX data set obtained with GAC 1 at an EBCT of 10 min. These comparisons were made for all other directly comparable data sets as shown in Figures S6–S9.

The comparisons illustrated in Figures 2 and S6–S9 demonstrate that neither PD- nor CD-RSSCT data can be directly used to predict PFAS breakthrough curves obtained at the pilot-scale. Comparisons of pilot-scale and CD-RSSCT breakthrough curves illustrated a close match in ${\text{BV}}_{50\%}$, but adsorption kinetics were dissimilar, with the majority of PFAS breakthrough curves obtained with CD-RSSCTs exhibiting a shallower slope than those obtained in the corresponding pilot columns (Figures 2 and S6–S9). These results imply that intraparticle diffusion of PFAS is not completely independent of GAC particle size when scaling factors are used that are larger than those resulting from comparisons of RSSCTs conducted with different particle sizes (see Section 3.1). In contrast to CD-RSSCTs, PD-RSSCTs overpredicted the onset of breakthrough and ${\text{BV}}_{50\%}$ (Figures 2 and S6–S9), a result that is consistent with those of Kempisty et al. (2022) for PFAS and others for neutral organic micropollutants (e.g., pharmaceuticals, personal care products, and herbicides; Kennedy et al., 2017; Summers et al., 2014). It is unclear why ${\text{BV}}_{50\%}$ for the PD-RSSCTs differed from those obtained in pilot studies and CD-RSSCTs. In the current study, PD-RSSCTs and CD-RSSCTs were conducted with the same particle sizes, suggesting that PD-RSSCTs with their lower HLRs (Table S4) underestimate the negative impact of DOM on PFAS removal by GAC. This observation may seem surprising because PD-RSSCTs effectively describe breakthrough curves for DOM surrogates such as TOC and UV254 absorbance (Summers et al., 1995). However, only a small fraction of the DOC competes with organic micropollutants for adsorption sites (Graham et al., 2000), suggesting that the DOM fractions affecting PFAS adsorption differ in their adsorption behavior from bulk DOM.

Overall, the results shown in Figures 2 and S6–S9 highlight that a scale-up approach is needed to predict PFAS removal in pilot- or full-scale GAC adsorbers from CD- or PD-RSSCT data.

### 3.3 | Scale-up of Freundlich adsorption capacity parameter (K)

**FIGURE 3:**
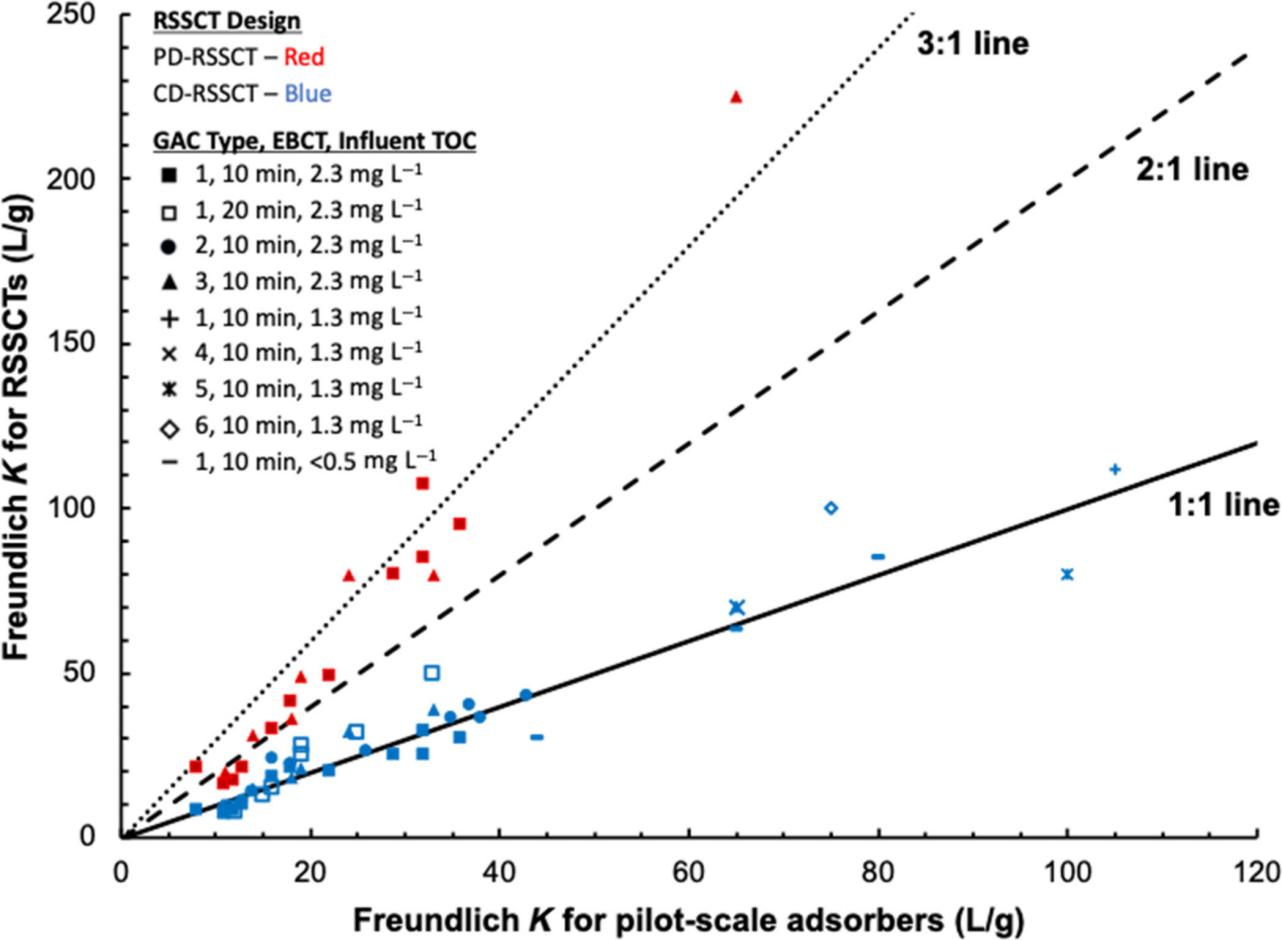
Comparison of Freundlich K values obtained in pilot studies and in corresponding constant diffusivity- and proportional diffusivity-rapid small-scale column tests.

To compare PFAS adsorption capacities across all pilot-scale and RSSCT data sets included in this study, experimental data were described with the PSDM to derive Freundlich adsorption capacity parameters (K; Tables S9–S13) as described in Section 2.2.4. K values obtained for RSSCTs were compared to corresponding pilot-scale values in Figure 3. Comparisons were made for 11 PFAS with different head groups and chain lengths, including novel PFEAs, six GAC products, and three background water matrices, including coagulated surface water, groundwater, and wastewater-impacted groundwater.

As shown in Figure 3, K values obtained for CD-RSSCT data sets effectively matched those of corresponding pilot tests for the range of studied PFAS, GACs, and water matrices. In contrast, Freundlich K values for PD-RSSCTs exceeded those derived from pilot-scale adsorbers by factor of ~1.5–3.5 (Figure 3). PD-RSSCT results obtained here agree with those of Kempisty et al. (2022), where PD-RSSCTs overestimated bed volumes to 10% and 50% breakthrough in full-scale GAC adsorbers treating groundwater by a factor of 1.4–2.1. The results for PFAS are also similar to those obtained for pesticides, pharmaceuticals, and other unregulated organic micropollutants (Kennedy et al., 2017; Summers et al., 2014). A scale-up approach involving a fouling index similar to that developed by Corwin and Summers (2010) could be used to scale up PD-RSSCT data to predict pilot-scale PFAS adsorption capacities. However, PD-RSSCTs are time consuming (>3 months run time) and require large volumes of water (>200 L) to achieve breakthrough for many PFAS. CD-RSSCTs require less time (3 days) and water (~20 L) compared to PD-RSSCTs to achieve breakthrough for the targeted PFAS. The shorter run time and lower water requirement suggests the CD-RSSCT design provides a promising basis for developing a scale-up approach to rapidly predict pilot- or full-scale PFAS breakthrough. Another advantage of CD-RSSCTs is that the Freundlich adsorption capacity parameter K and ${\text{BV}}_{50\%}$ can be directly predicted, that is, no fouling index is needed for scale-up (Figure 3).

### 3.4 | Scaling adsorption kinetics to predict pilot-scale breakthrough

As shown in Figures 2 and S6–S9, PFAS breakthrough curves obtained with CD-RSSCTs tend to be somewhat flatter than those obtained in corresponding pilot studies. In other words, the intraparticle diffusion of PFAS is not independent of GAC particle size, as assumed in the CD-RSSCT design. The development of a scale-up approach for predicting pilot-scale breakthrough will therefore require transforming the CD-RSSCT data to generate breakthrough profiles that account for a change in intraparticle diffusivity as GAC particle size changes. The PSDM can be used to determine the dependence of the intraparticle diffusivity (or intraparticle diffusive flux) on GAC particle size via the normalized total intraparticle flux (NTIF, Summers et al., 2014).

Three scenarios are possible for the NTIF, as outlined in Table S14. The NTIF has a value of 1 for the base case (maximum pore diffusion flux case in Table S14), when surface diffusion is negligible and the pore diffusion coefficient is equal to the liquid diffusion coefficient, meaning tortuosity (τ) is 1. When the diffusion path is more tortuous (path length exceeds that of a straight line), tortuosity increases and NTIF, which is calculated as the inverse of tortuosity, decreases (pore diffusion flux case in Table S18). If, on the other hand, intraparticle diffusion occurs at a rate exceeding the maximum pore diffusion flux case, surface diffusion is invoked, and the NTIF is calculated as the sum of the pore and surface diffusion flux contributions (pore plus surface diffusion flux case in Table S14).

To address the particle size-dependence of intraparticle diffusion, first kinetic parameters for individual breakthrough curves of the CD-RSSCT and pilot-scale data sets were obtained. For example, the best fit of the PSDM for the GenX breakthrough data obtained with the CD-RSSCT (Figure 2) was achieved for a tortuosity (τ) of 2 and a surface-to-pore diffusion flux ratio (SPDFR) of 10^−30^ (Table S9). Therefore, ${\text{ NTIF }}_{\text{RSSCT }}$ for GenX falls into the category of pore diffusion flux (Table S14) and was calculated to be 0.5. The NTIF for the pilot-scale can then be determined from the NTIF of the CD-RSSCT using Equation (3).

(1) $${\text{NTIF}}_{\text{pilot-scale }}={\left(\text{SF}\right)}^{X}\times {\text{NTIF}}_{\text{RSSCT}},$$

where the scaling factor (SF) represents the ratio of the GAC particle diameters used in the pilot-scale and CD-RSSCT $\left(\text{SF}=d_{\text{pilot }}/{\text{d}}_{\text{RSSCT }}\right)$ and the value of X represent proportionality factor, where a value of 0 represents constant diffusivity and a value of 1 represents proportional diffusivity.

To obtain the value of X in Equation (3), the pilot-scale and CD-RSSCT data sets shown in Figures S6–S9 were described with the PSDM to determine NTIF values. Using the scaling factors shown in Table S4, a median value of 0.27 was obtained for X, with a range of 0 to 0.69 (Figure S10). For user-friendliness, the validation approach moving forward used a proportionality constant of X = 0.25, which is sufficiently close to the median value of 0.27.

**FIGURE 4:**
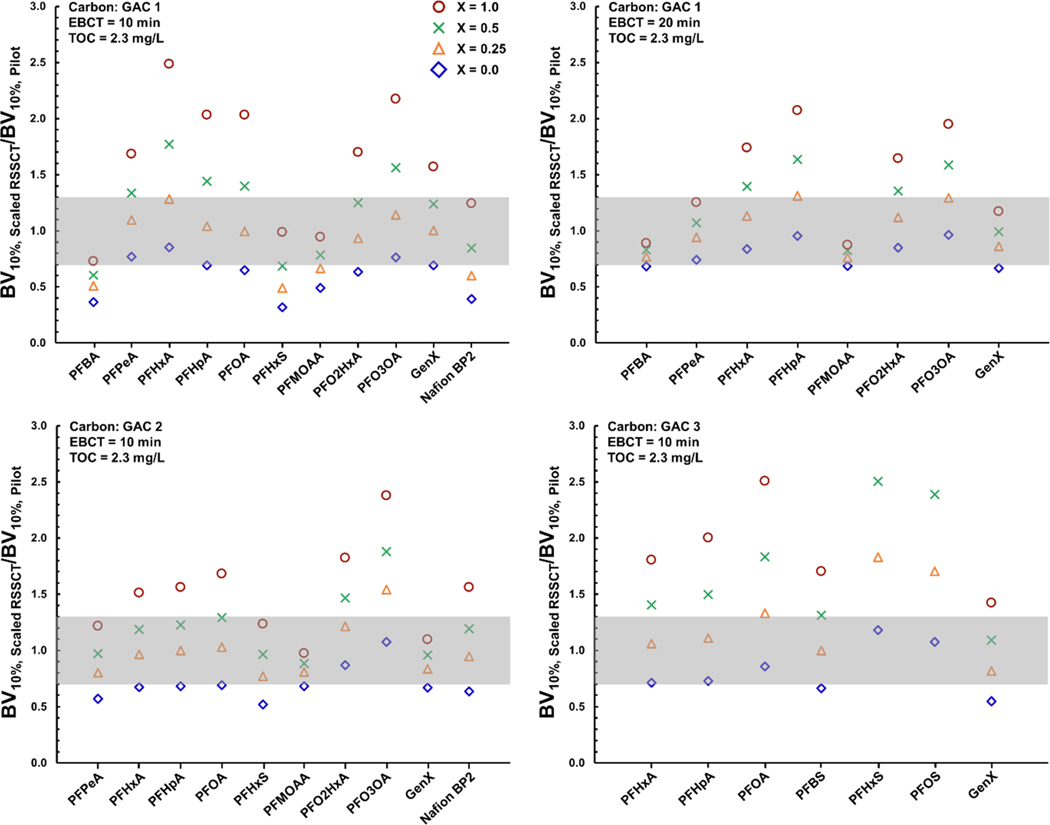
Comparison of bed volumes to 10% breakthrough for pilot-scale and scaled-up constant diffusivity-rapid small-scale column test data. Shaded region represents ±30% variance around perfect agreement.

The sensitivity of the particle-size dependence of the intraparticle diffusivity was further investigated by calculating ${\text{NTIF}}_{\text{pilot-scale }}$ from ${\text{ NTIF }}_{\text{RSSCT }}$ with Equation (3) with proportionality factors of 0, 0.25, 0.5, and 1. For each proportionality factor, ${\text{NTIF}}_{\text{pilot-scale }}$ values and associated kinetic parameters were calculated using the approach highlighted in Table S15, and resulting Freundlich capacity and adsorption kinetic parameters resulting for the different proportionality factors are summarized in Tables S16–S21 and compared to the parameters that resulted in the best PSDM fit for the pilot data. Scaled-up breakthrough curves for each PFAS were subsequently predicted with the Freundlich K value obtained from the CD-RSSCT and the scaled kinetic parameters that served as input parameters to the PSDM. Bed volumes to 10, 20, 50, and 70% breakthrough for scaled-up breakthrough curves were then compared to the bed volumes of water that could be treated at the pilot-scale to reach the same level of breakthrough for each proportionality case (X = 0, 0.25, 0.5, 1; Figure 4 for 10% breakthrough and Figures S11–S13 for 20%, 50%, and 70% breakthrough).

Results in Figure 4 and Table S22 highlight that 75% of CD-RSSCT results scaled with a proportionality factor of X = 0.25 were within 30% of the bed volumes of water that could be treated to 10% breakthrough (${\text{BV}}_{10\%}$) in the pilot study. This result aligns with the calculated median for proportionality factors using Equation (3). For the other three scaling cases that were considered (X = 0, 0.5, and 1.0), only 42%, 47%, and 33% of the scaled-up data, respectively, fell within 30% of the ${\text{BV}}_{10\%}$ value determined in the pilot study. Results shown in Figure 4 were obtained with 3 GACs and 2 EBCTs (for GAC1), suggesting that changing the GAC type or EBCT did not affect the scale-up approach. Comparisons for bed volumes to 20% breakthrough (${\text{BV}}_{20\%}$) demonstrated that 75% of the CD-RSSCT data scaled up with X = 0.25 fell within 30% of the pilot-scale data compared to 72%, 58%, and 36% for X = 0, 0.5, and 1.0, respectively (Table S23 and Figure S11). At higher breakthrough percentages, ${\text{BV}}_{50\%}$ and ${\text{BV}}_{70\%}$ values were relatively insensitive to the proportionality factor (Figures S12 and S13); for X = 0.25, 78% and 75% of the scaled-up RSSCT values were within 30% of the pilot-scale ${\text{BV}}_{50\%}$ and ${\text{BV}}_{70\%}$ values, respectively (Tables S24 and S25). Applying the proportionality factor of X = 0.25 in Equation (3) to scale up all CD-RSSCT data obtained in Water A, PSDM predictions effectively matched pilot-scale breakthrough data for most PFAS (Figures S14–S17). It is important to note that in some cases, the RSSCT data that were scaled up with X = 0.25 fell outside of the uncertainty envelopes (Figures 4 and S12–S17), especially for (1) PFAS, for which no mass-labeled internal standards were available at the time of data collection (e.g., PFMOAA, PFO2HxA, PFO3OA, Nafion by-product 2) and (2) PFAS that were present at very low levels in the pilot column influent, which likely added uncertainty to breakthrough data, especially for 10% and 20% breakthrough (e.g., PFHxS). A limitation of the presented scale-up approach is that neither CD-RSSCT data nor the pseudo-single-solute modeling approach simulates chromatographic effects that lead to PFAS concentrations in the GAC effluent exceeding those in the GAC influent, which is commonly observed when the adsorption capacity of GAC for short-chain PFAS becomes exhausted (Figures S3–S5 and S14–S17).

### 3.5 | Validation of scale-up approach

To validate the scale-up approach developed for Water A, it was applied to data sets obtained with groundwater (Water B, TOC <0.5 mg/L) and wastewater-impacted groundwater (Water C, TOC = 1.3–1.6 mg/L). In the pilot study conducted with Water B, breakthrough curves were determined for PFBA, PFPeA, and PFHxA with GAC A. For Water C, PFOA breakthrough data were obtained for four GACs. All breakthrough curves obtained at the pilot-scale and in corresponding CD-RSSCTs were described with the PSDM (Tables S20 and S21). The scale-up approach was validated by comparing (1) Freundlich K values (Figure 3) and (2) ${\text{BV}}_{10\%}$, ${\text{BV}}_{20\%}$, ${\text{BV}}_{50\%}$, and ${\text{BV}}_{70\%}$ values (Figures S18–S21). In Figure 3, Freundlich K values from the CD-RSSCTs conducted in Water B and Water C were in good agreement with those derived from the pilot-scale data. Additionally, scaling intraparticle diffusion using a proportionality factor of X = 0.25, as described in Section 3.4, led to predicted values of ${\text{BV}}_{10\%}$, ${\text{BV}}_{20\%}$, ${\text{BV}}_{50\%}$, and ${\text{BV}}_{70\%}$ that typically matched pilot-scale values within 30% (Figures S18–S21). These results suggest that the scale-up approach is valid for PFAS breakthrough percentages of up to 70% across a wide range of background water matrices, including coagulated, settled surface water, groundwater, and wastewater-impacted groundwater.

**FIGURE 5:**
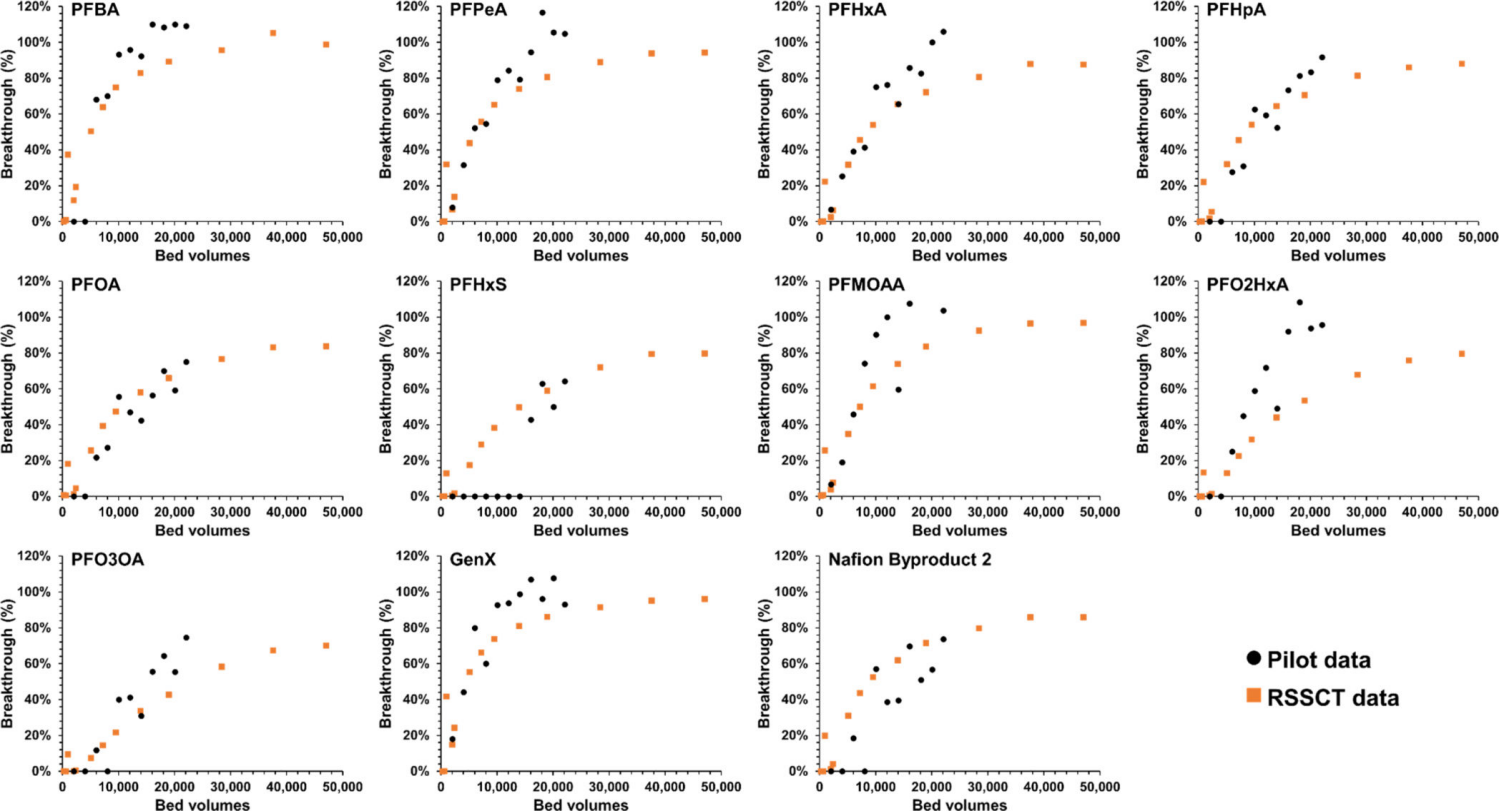
Direct comparison of per- and polyfluoroalkyl substances breakthrough curves obtained with a rapid small-scale column test designed with X = 0.25 (Equation 1) and in a corresponding pilot study treating Water A (TOC = 2.3 mg/L), Carbon: granular activated carbon 1, simulated empty bed contact time: 10 min.

The scale-up approach described so far is based on conducting CD-RSSCTs and scaling up resulting breakthrough data using the PSDM with a proportionality factor (X) of 0.25. Alternatively, the proportionality factor of X = 0.25 could be directly applied to the RSSCT design equation (Equation 1). To explore this scale-up alternative, which would allow for a direct comparison of RSSCT and pilot-scale data (i.e., the PSDM would not be needed to scale up RSSCT data), an RSSCT for GAC 1 was designed according to Equation (1) with a proportionality factor of X = 0.25. Resulting RSSCT data generally matched pilot-scale data well (Figure 5) although discrepancies can be observed at high breakthrough percentages (generally >70%). Differences were more pronounced for PFMOAA, PFO2HxA, and Nafion by-product 2, for which matching mass-labeled internal standards were not available at the time of data collection. Overall, results in Figure 5 suggest that an RSSCT designed with a proportionality value of X = 0.25 yields PFAS breakthrough data that can directly predict pilot-scale (and hence full-scale) data up to 70% breakthrough. A caveat is that RSSCTs are conducted with a single batch of water and do not capture seasonal variability in source water quality and/or hydraulic loading rates.

## 4 | CONCLUSIONS

An approach is presented that permits the prediction of PFAS breakthrough curves in pilot-and full-scale GAC adsorbers from RSSCT data. The scale-up approach was developed and validated with pilot data obtained with three water types (coagulated/settled surface water [TOC = 2.3 mg/L], groundwater [TOC <0.5 mg/L], and wastewater-impacted groundwater [TOC = 1.3–1.6 mg/L]), six GACs (three reagglomerated, bituminous coal-based; coconut shell-based; lignite-based; direct activated subbituminous-based), two EBCTs, and 11 PFAS, including PFCAs, PFSAs, and PFEAs. Key findings were that (1) intraparticle diffusivity of PFAS was dependent on GAC particle diameter $\left(d_{\text{p}}\right)$ and proportional to $\left(d_{\text{p}}\right)$^0.25^, when considering data up to about 70% PFAS breakthrough, and (2) ${\text{BV}}_{50\%}$ values for CD-RSSCTs as well as for an RSSCT conducted with a proportionality factor (X) of 0.25 closely matched ${\text{BV}}_{50\%}$ values obtained in pilot-scale studies such that a fouling index (Corwin & Summers, 2010) is not needed for scale-up. Two options for collecting RSSCT data are recommended: (1) conduct CD-RSSCTs, which permit rapid completion of the data collection effort (for a scaling factor of 13.3, which was primarily used in this study, 1 day of CD-RSSCT operation corresponds to ~177 days of pilot- or full-scale operation); however, breakthrough curves resulting from CD-RSSCTs require adjustment with the PSDM to account for the particle size-dependence of intraparticle diffusivity or (2) conduct RSSCTs designed with a proportionality factor of X = 0.25, which require a longer completion time (for a scaling factor of 13.3, 1 day of RSSCT operation corresponds to 93 days of pilot- or full-scale operation), but resulting PFAS breakthrough curves can be used directly to predict pilot- or full-scale performance for PFAS breakthrough percentages of up to 70%. It should be noted that scale-up approaches were validated for scaling factors in the range of 7.2–15.4 in this study; additional work should be conducted to assess whether scaling factors outside of this range are suitable. Other opportunities for future research include expanding the range of (1) DOM types and DOC concentrations, (2) GAC types, and (3) PFAS subclasses. In this study, commonly occurring anionic PFAS such as PFCAs with 4–8 carbon atoms and PFSAs with 4, 6, and 8 carbon atoms (limited data) as well as less well understood PFEAs (GenX, PFMOAA, PFO2HxA, PFO3OA, Nafion by-product 2) were included, but many other PFAS subclasses, such as neutral, cationic, and zwitterionic PFAS exist, for which the presented scale-up approach has not been validated. On a more fundamental level, research is needed to understand why the adsorption capacity of organic micropollutants, including PFAS, differs between CD-RSSCTs, PD-RSSCTs, and pilot-scale studies. Bench- and pilot-scale experiments are therefore needed that assess the effect of HLR on the uptake capacity of organic micropollutants in the presence of DOM. Recently, Cheng and Knappe (2024) showed that HLR is an important factor in determining the PFAS uptake capacity of anion exchange resins when treating water containing DOM. Furthermore, it is important to understand intraparticle adsorbate distributions from experiments conducted with different HLRs at different scales to gain a better understanding of how GAC fouling and HLR are linked. While there are a range of outstanding applied and fundamental needs, we expect the presented RSSCT design and scale-up protocols will be effective for supporting the development of cost estimates and the design of GAC treatment processes for PFAS removal.

## Supplementary Material

Supinfo

## Article Impact Statement

Protocols were developed for conducting rapid small-scale column tests and scaling-up resulting breakthrough data to predict per- and polyfluoroalkyl substances removal in full-scale granular activated carbon adsorbers.
