# Supplementary material for: Predicting per- and polyfluoroalkyl substances removal in pilot-scale granular activated carbon adsorbers from rapid small-scale column tests
Source: AWWA Water Sci. Author manuscript; Available in PMC 2025 Mar 19. (PMC11706541; doi:10.1002/aws2.1369)
Supplement: Supinfo [file NIHMS1976884-supplement-Supinfo.pdf]

SUPPORTING INFORMATION

PREDICTING PFAS REMOVAL IN PILOT-SCALE GRANULAR ACTIVATED CARBON ADSORBERS FROM RAPID SMALL-SCALE COLUMN TESTS

Zachary R. Hopkins, Detlef R.U. Knappe

Department of Civil, Construction, and Environmental Engineering, North Carolina State University, Raleigh, North Carolina, United States

Text S1. Rapid small-scale column tests

The rapid small-scale column test (RSSCT) is an approach to reduce the time and cost of determining full-scale GAC performance data (Crittenden et al. 1986; Crittenden et al. 1987; Summers et al. 1995). The design of RSSCTs is based on the principle of similitude and the dimensionless form of the pore surface diffusion model (PSDM). RSSCTs use a smaller GAC particle size than the as-received size used in pilot-scale or full-scale adsorbers. To maintain perfect similitude between the RSSCT and pilot- or full-scale adsorbers, the dimensionless parameters shown in **Table S1.1** need to be kept constant.

Table S1.1. Dimensionless parameters obtained from the pore surface diffusion model

Parameter	Equation
Pore solute distribution parameter	$D_g = \dfrac{\varepsilon_p(1-\varepsilon)}{\varepsilon}$
Pore diffusion modulus	$Ed_p = \dfrac{4D_pD_gL\varepsilon}{d_p^2 v_f}$
Surface diffusion modulus	$Ed_s = \dfrac{4D_sC_FL\varepsilon}{d_p^2 v_f}$
Peclet number	$Pe = \dfrac{Lv_f}{\varepsilon D_z}$
Stanton number	$St = \dfrac{2k_fL(1-\varepsilon)}{d_p v_f}$

intraparticle porosity (ε_p), bed porosity (ε), pore diffusion coefficient (D_p), particle diameter (d_p), surface diffusion coefficient (D_s), capacity factor ($C_F = q_o\rho_b/C_o\varepsilon$), bed length (L), hydraulic loading rate (v_f), dispersion coefficient (D_z), film mass transfer coefficient (k_f), bed density (ρ_b), solid phase concentration (q_0) in equilibrium with influent concentration (C_0).

Using the principle of similitude, RSSCT design equations have been developed from the dimensionless parameters shown in Table S1.1 using the assumptions that intraparticle diffusivity is either independent of GAC particle size [constant diffusivity (CD) RSSCT design, Crittenden et al. 1986] or proportional to GAC particle size [proportional diffusivity (PD) RSSCT design, Crittenden et al. 1987]. RSSCT design equations are summarized in **Table S1.2**. **Figure S1.1** shows the set-up for a CD-RSSCT.

To prepare GAC beds for RSSCTs, the desired mass of dry GAC was weighed, transferred to a small glass beaker, and soaked overnight in ultrapure water (UPW) in a vacuum desiccator. Using a glass Pasteur pipet, pre-wetted GAC was subsequently transferred to polypropylene tubing that contained quartz wool support (**Figure S1.1**) and was partially filled with UPW. The

outside of the RSSCT tubing was lightly tapped to compact the GAC bed until the desired bed depth, which was calculated from the packed bed density of the GAC, was reached. Wall effects were avoided by assuring that the ratio of the GAC column diameter to the GAC particle diameter exceeded the minimum requirement of 8-10 (e.g., Chu and Ng 1989); in all cases, the ratios were >40 (**Tables S4-S7**). During CD-RSSCT experiments, no problems were encountered with pressure buildup, likely because (1) influent water was prefiltered, (2) the hydraulic loading rate was low, and (3) run times were relatively short (~3 days).

Table S1.2. RSSCT design equations

Parameter	Equation
Scaling factor (SF)	$SF = \left(\frac{d_{p,LC}}{d_{p,SC}}\right)$
Diffusion coefficient (D)	$D_{SC} = (SF)^{-X} D_{LC}$
Empty bed contact time (EBCT)	$EBCT_{SC} = (SF)^{X-2} EBCT_{LC}$
Design factor (DF)	$DF = \frac{EBCT_{SC}}{EBCT_{LC}} = (SF)^{X-2}$
Hydraulic loading rate (HLR)	$HLR_{SC} = HLR_{LC} \left(\frac{d_{LC}}{d_{SC}}\right)\left(\frac{Re_{SC,min}}{Re_{LC}}\right)$

d – GAC particle size; X – proportionality factor (X = 0 for CD-RSSCTs, X = 1 for PD-RSSCTs); Re – Reynolds number, $Re_{SC,min}$ is the minimum Reynolds # for the RSSCT column, and criteria for $Re_{SC,min}$ are shown in **Table S1.3**

Table S1.3. Literature criteria for minimum Reynolds number

Product of Re-Sc	Minimum Re	Reference
150	0.13	Crittenden et al. 1989
200	0.17	Berrigan 1985
500	0.42	Summers et al. 2014
1000	0.85	Berrigan 1985

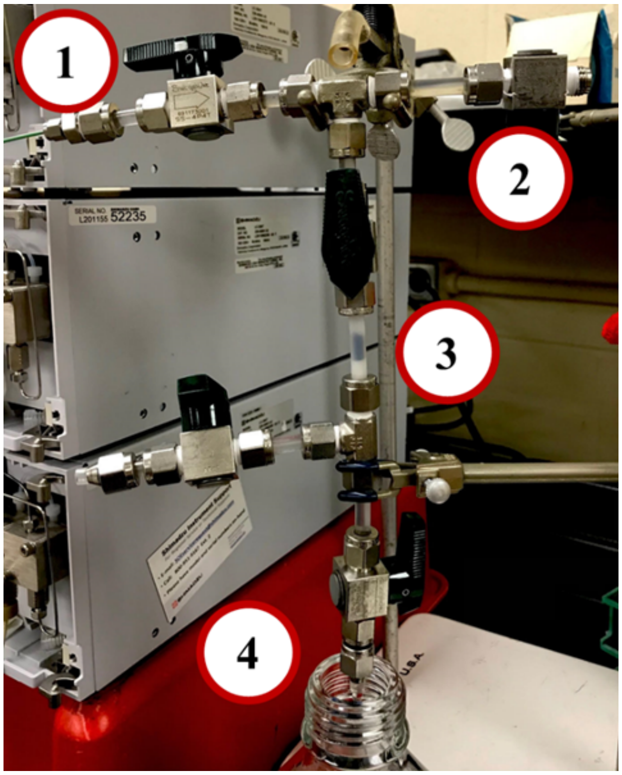

Figure S1.1. Experimental set-up for constant diffusivity RSSCT. The influent is delivered from the feed container to the column with an HPLC pump (1). Influent sampling port (2). Each column is filled with designed mass of crushed GAC (3). Treated water is sampled for PFAS and TOC analysis post treatment from a sampling port (4).

Text S2. PFAS Analysis

Materials

All materials used for sample collection and analysis have been previously checked for their suitability for PFAS analysis and were found to not contribute to measurable additions or losses of PFASs. The following materials were used for sample preparation and analysis:

a. 15 mL polypropylene conical centrifuge tubes with polypropylene screw caps (Corning Falcon P/N 352097)
b. Genesee Scientific, Reach Olympus Classic Pipet Tips, plastic, 1-10 µL capacity (P/N 24-120R), checked to be PFAS-free
c. Genesee Scientific, Olympus Class Pipet Tips, plastic, 10-200 µL capacity (P/N 24-151R), checked to be PFAS-free
d. Genesee Scientific, Reach Olympus Pipet Tips, plastic, 100-1000 µL capacity (P/N 24-165R), checked to be PFAS-free
e. Eppendorf epTIPS Pipette tips, plastic, 50-1000 µL capacity (P/N 022492055), checked to be PFAS-free
f. Thermo Scientific™ 9mm Plastic Screw Thread Vials, 2 mL capacity (P/N 03-376-900)
g. Thermo Scientific™ 9 mm Autosampler Vial Screw Thread Caps (P/N 03-376-483)
h. 24-component native PFAS standard mix (2.0 µg/mL in methanol), Wellington Laboratories P/N PFAC-24PAR
i. Individual PFEA standards (HFPO-DA, ADONA, F-53B major) (50.0 µg/mL in methanol), Wellington Laboratories P/N HFPO-DA, NaDONA, 9Cl-PF3ONS
j. Individual PFECA/PFESA/PFES-CA standards (1 mg/mL in water), Chemours (not commercially available)
k. 19-component mass-labelled PFAS standard mix (1.0 µg/mL in methanol), Wellington Laboratories P/N MPFAC-24ES
l. Mass-labelled HFPO-DA (50 µg/mL in methanol), Wellington P/N M3HFPO-DA

Sample preparation

a. Using clean pipette tips, transfer 1,620 µL of sample from the 15-mL centrifuge tube to a new autosampler vial and add 180 µL of the internal standard mix.
b. Cap the vial with a screw cap and vortex the vial for 10 seconds.

The same sample preparation procedure was applied to method blanks, calibration standards, and quality control samples.

Liquid Chromatography-Tandem Mass Spectrometry (LC-MS/MS)

PFAS analysis was performed by high performance liquid chromatography (1260 series, Agilent) and tandem mass spectrometry (Ultivo, Agilent). Two separate injections were completed for

each sample at high and low source temperature settings to maximize responses for the targeted PFAS.

Liquid chromatography method

A 800-μL aliquot of each sample, calibration standard, and quality control sample was injected into the LC system that was equipped with a 900 μL sample loop. Analytes were chromatographically separated using a Zorbax RR Eclipse Plus C18 column (4.6 x 50 mm, 3.5 μm; Agilent). Conditions were as follows: eluent flow rate: 0.7 mL/min; column temperature: 50 °C; mobile phase A: ammonium acetate buffer (5 mM) in water, and mobile phased B: methanol; gradient: 0-18 min linear from 90:10 A/B to 5:95 A/B, 18-22 min constant 5:95 A/B; followed by a 6 min post-analysis time for equilibration.

Mass spectrometer settings

PFAS were detected using electrospray ionization in negative polarity mode and multiple reaction monitoring. Ion source parameters are provided in **Table S2.1.** For quantification of PFAS containing a carboxylic acid moiety, low-temperature settings were used. For quantification of PFAS with a sulfonic acid moiety, high-temperature settings were used.

Table S2.1. Mass spectrometer ion source parameters

Parameter	Low Temperature	High Temperature
Drying gas temperature (°C)	100	230
Drying gas flow rate (L/min)	13	13
Sheath gas temperature (°C)	250	350
Sheath gas flow rate (L/min)	12	12
Nebulizer pressure (psi)	20	15

PFAS quantitation

Data acquisition and processing were performed using Agilent MassHunter Quantitative Analysis Version B.09.00. A list of target analytes and internal standards used for quantitation is provided in **Table S2.2** With the exception of PFBA, PFPeA, PFMOAA, and PMPA each analyte has a pair of precursor-product ion transitions for quantitation and confirmation, respectively. Quantitation was conducted using an isotope dilution approach; i.e., the ratio of the peak area of the analyte to the peak area of the internal standard was used to develop standard curves and determine analyte concentrations. If a mass-labeled analog of the analyte was not available, a structurally similar mass-labeled internal standard with a similar retention time was used for quantitation (**Table S2.2**).

Table S2.2. PFAS analyte acronyms, precursor and product ion m/z values, fragmentor and collision energies, and capillary voltages – (1) quantitation ion, (2) confirmation ion (note: only one transition is available for PFBA and PFPeA)

Name (Acronym)	Precursor Ion	Confirmation Ion	Fragmentor (V)	Collision Energy (V)	Capillary Voltage (V)	Internal standard
**High Temperature Ion Source Compounds**						
NVHOS (1)	296.9	79.9	140.0	48.0	1500	MPFBS
NVHOS (2)	269.9	135.1	140.0	24.0	1500	MPFBS
Nafion Byproduct 4 (1)	440.9	197.0	140.0	32.0	1500	MPFBS
Nafion Byproduct 4 (2)	440.9	241.0	140.0	20.0	1500	MPFBS
Nafion Byproduct 2 (1)	462.9	263.0	140.0	24.0	2000	MPFHxS
Nafion Byproduct 2 (2)	462.9	213.0	140.0	32.0	2000	MPFHxS
6:2FTS (1)	427.0	406.9	160.0	20.0	2000	M6:2 FTS
6:2 FTS (1)	427.0	80.9	160.0	40.0	2000	M6:2 FTS
PFBS (1)	298.9	79.9	140.0	40.0	1500	MPFBS
PFBS (2)	298.9	98.8	140.0	28.0	1500	MPFBS
PFHxS (1)	398.9	79.8	166.0	68.0	2000	MPFHxS
PFHxS (2)	398.9	98.9	166.0	36.0	2000	MPFHxS
PFOS (1)	498.9	79.7	170.0	76.0	2000	MPFOS
PFOS (2)	498.9	98.8	170.0	44.0	2000	MPFOS
**Low Temperature Ion Source Compounds**						
PFBA (1)	213.0	169.0	84.0	0.0	1500	MPFBA
PFPeA (1)	263.0	219.0	80.0	0.0	1500	MPFPeA
PFHxA (1)	313.0	268.9	94.0	0.0	2000	MPFHxA
PFHxA (2)	313.0	119.0	94.0	16.0	2000	MPFHxA
PFHpA (1)	363.0	319.0	100.0	0.0	2000	MPFHpA
PFHpA (2)	363.0	169.1	100.0	8.0	2000	MPFHpA
PFOA (1)	413.0	369.0	103.0	0.0	2000	MPFOA
PFOA (2)	413.0	168.9	103.0	12.0	2000	MPFOA
PFNA (1)	463.0	418.9	100.0	0.0	2000	MPFNA
PFNA (2)	463.0	219.0	100.0	8.0	2000	MPFNA
PFDA (1)	513.0	469.0	120.0	0.0	4000	MPFDA
PFDA (2)	513.0	269.0	120.0	8.0	4000	MPFDA
PFMOAA (1)	179.0	85.0	79.0	4.0	1500	MPFBA
PFMOAA (2)	179.0	135.0	79.0	0.0	1500	MPFBA
PMPA (1)	229.0	184.9	89.0	0.0	1500	MPFBA
PMPA (2)	229.0	85.1	89.0	16.0	1500	MPFBA
PEPA (1)	235.0	135.0	100.0	16.0	1500	MGenX
PEPA (2)	279.0	235.0	100.0	0.0	1500	MGenX
GenX (1)	285.0	169.0	108.0	0.0	2000	MGenX
GenX (2)	329.0	169.1	108.0	4.0	2000	MGenX
PFO2HxA (1)	245.0	85.0	95.0	0.0	1500	MGenX
PFO2HxA (2)	201.0	85.0	95.0	0.0	1500	MGenX

PFO3OA (1)	311.0	85.0	105.0	0.0	2000	MGenX
PFO3OA (2)	311.0	151.0	105.0	0.0	2000	MGenX
HydroEve (1)	427.0	283.0	100.0	4.0	2000	MGenX
HydroEve (2)	427.0	262.9	100.0	12.0	2000	MGenX
PFO4DA (1)	376.9	85.0	110.0	0.0	2000	MGenX
PFO4DA (2)	376.9	150.9	110.0	0.0	2000	MGenX
PFO5DoA (1)	442.9	84.8	110.0	0.0	2000	MGenX
PFO5DoA (2)	442.9	150.8	110.0	0.0	2000	MGenX
**Internal Standards**						
MPFBA	217.0	172.0	85.0	0.0	1500	
MPFPeA	268.0	223.0	55.0	0.0	1500	
MPFHxA	318.0	273.0	95.0	0.0	2000	
MPFHpA	367.0	322.0	60.0	0.0	2000	
MPFOA	421.0	376.0	105.0	0.0	2000	
MPFNA	472.0	427.0	105.0	0.0	2000	
MFPDA	519.0	474.0	110.0	0.0	4000	
MGenX	287.0	169.0	108.0	0.0	2000	
MPFBS	302.0	79.9	120.0	40.0	1500	
MPFHxS	402.0	81.0	170.0	56.0	2000	
MPFOS	507.0	81.0	190.0	60.0	2000	
M6:2FTS	429.0	409.0	165.0	20.0	2000	

Table S1. Average PFAS concentrations for RSSCT and pilot tests in surface water and groundwater.

PFAS	Concentration (ng/L)					
	Water A		Water B		Water C	
	Coagulated surface water		Groundwater		Wastewater-impacted Groundwater	
	Pilot	RSSCT	Pilot	RSSCT	Pilot	RSSCT
Perfluoroalkylcarboxylic acids						
Perfluorobutanoic acid (PFBA)	10 (± 2)	109 (± 2)	9	129 (± 1)	--	--
Perfluoropentanoic acid (PFPeA)	21 (± 7)	112 (± 2)	13	133 (± 0.5)	--	--
Perfluorohexanoic acid (PFHxA)	23 (± 9)	119 (± 36)	19	129 (± 0.2)	2.8 (± 0.3)	--
Perfluoroheptanoic acid (PFHpA)	16 (± 9)	106 (± 1)	10	106 (± 0.8)	--	--
Perfluorooctanoic acid (PFOA)	11 (± 2)	98 (± 3)	33	143 (± 2)	16 (± 2)	15
Perfluorononanoic acid (PFNA)	--	106 (± 4)	44	157 (± 2)	2.2 (± 0.75)	--
Perfluorodecanoic acid (PFDA)	--	121 (± 13)	--	136 (± 2)	3.1 (± 0.57)	--
Perfluoroalkylsulfonic acids						
Perfluorobutane sulfonate (PFBS)	3.0 (± 0.7)	118 (± 2)	--	129 (± 2)	14 (± 1.3)	--
Perfluorohexane sulfonate (PFHxS)	5.0 (± 1.0)	119 (± 1)	2	98 (± 0.1)	11 (± 0.88)	--
Perfluorooctane sulfonate (PFOS)	12 (± 2)	131 (± 8)	6	139 (± 0.1)	23 (± 1.8)	--
Fluorotelomer sulfonic acid						
6:2 fluorotelomer sulfonic acid (6:2 FTS)	--	89 (± 2)	--	114 (± 0.02)	--	--
Per- and polyfluoroalkylether carboxylic acids						
Perfluoro-2-methoxyacetic acid (PFMOAA)	1450 (± 700)	158 (± 1)	--	85 (± 1)	--	--
Perfluoro-2-methoxypropanoic acid (PMPA)	--	111 (± 2)	--	107 (± 2)	--	--
Perfluoro-2-ethoxypropanoic acid (PEPA)	--	100 (± 0.3)	--	91 (± 0.3)	--	--
Perfluoro-2-propoxypropanoic acid (GenX)	19 (± 14)	94 (± 1)	--	132 (± 1)	--	--
Perfluoro-3,5-dioxahexanoic acid (PFO2HxA)	116 (± 66)	119 (± 2)	--	94 (± 0.7)	--	--
Perfluoro-3,5,7-trioxaoctanoic acid (PFO3OA)	52 (± 30)	107 (± 2)	--	112 (± 0.4)	--	--
Perfluoro-3,5,7,9-tetraoxadecanoic acid (PFO4DA)	24 (± 10)	97 (± 2)	--	96 (± 2)	--	--
Perfluoro-3,5,7,9,11-pentaoxadodecanoic acid (PFO5DoA)	--	97 (± 7)	--	98 (± 7)	--	--
2,2,3,3-Tetrafluoro-3-{[1,1,1,2,3,3-hexafluoro-3-(1,2,2,2-tetrafluoroethoxy)propan-2-yl]oxy}propanoic acid (HydroEve Acid)	--	96 (± 1)	--	97 (± 1)	--	--

Polyfluoroalkylether sulfonic acids						
1,1,2,2-tetrafluoro-2-(1,2,2,2-tetrafluoro-ethoxy)ethane sulfonic acid (NVHOS)	--	93 (± 1)	--	106 (± 1)	--	--
Perfluoro-2-{[perfluoro-3-(perfluoroethoxy)-2-propanyl]oxy}ethanesulfonic acid (Nafion by-product 2)	33 (± 18)	99 (± 1)	--	103 (± 1)	--	--

Table S2. Water quality parameters for RSSCT and Pilot tests in surface water and groundwater.

**Source**	**Water A (Coagulated surface water)**				**Water B (Groundwater)**		**Water C (Wastewater-impacted groundwater)**	
**Scale**	**RSSCT**			**Pilot**	**RSSCT**	**Pilot**	**RSSCT**	**Pilot**
**TOC (mg L^{-1})**	1.3	1.5	2.3	2.3	<0.5	<0.5	1.6	1.3
**UV254 (cm^{-1})**	0.130 (±0.004)	0.138 (±0.003)	0.201 (±0.012)	--	0.008	--	--	--

Table S3. GAC types and characteristics for RSSCT and pilot-scale tests.

GAC	Base material	U.S mesh size		Apparent (bed) density (g/cm^3)	Pore volume (cm^3/g)		
		Pilot	RSSCT		Primary micropore (<8 Å)	Secondary micropore (8-20 Å)	Mesopore (20-50 Å)
1	Reagglomerated bituminous coal	12x40	200x230[a]<br>100x140[b]	0.54	0.1389	0.1532	0.0403
2	Reagglomerated bituminous coal	12x40	200x230[a]	0.50	0.1210	0.1838	0.0628
3	Coconut shell	12x30	200x230[a]	0.41	0.1770	0.3199	0.1114
4	Reagglomerated bituminous coal	12x40	100x140[b]	0.62	--	--	--
5	Lignite	10x30	80x120[b]	0.39	--	--	--
6	Direct activated subbituminous coal	12x40	100x140[b]	0.50	--	--	--

[a] mesh size used for Source Water A and Water B RSSCTs

[b] mesh size used for Source Water C RSSCTs

Table S4. Design specifications for pilot-scale GAC adsorbers and RSSCTs conducted with Water A

Parameter	GAC 1							GAC 2		GAC 3		
	Pilot		PD-RSSCT-PP	CD-RSSCT-SS	CD-RSSCT-PP[a]	CD-RSSCT-PP	XD-RSSCT-PP[b]	Pilot	CD-RSSCT-PP[a]	Pilot	PD-RSSCT-PP	CD-RSSCT-SS
TOC (mg L^{-1})	2.3	2.3	2.3	2.3	2.3	2.3	2.2	2.3	2.3	2.3	2.3	2.3
Media US Standard mesh size	12x40	12x40	200x230	200x230	200x230	200x230	200x230	12x40	200x230	12x30	200x230	200x230
Log-mean GAC particle diameter (mm)	0.92	0.92	0.069	0.069	0.069	0.069	0.069	0.92	0.069	1.06	0.069	0.069
Scaling factor	-	-	13.3	13.3	13.3	13.3	13.3	-	13.3	-	15.4	15.4
(Simulated) EBCT (min)	9.4	18.7	0.769 (10.3)	0.056 (10.1)	0.057 (9.7)	0.112 (20.1)	0.107 (10.2)	10.1	0.056 (9.9)	9.6	0.636 (9.8)	0.043 (10.1)
GAC mass (g)	5195	10390	0.808	0.0672	0.0427	0.0853	0.0815	4952	0.0395	4172	0.533	0.0514
Bed diameter (mm)	102	102	4.76	4.00	3.18	3.18	3.18	102	3.18	102	4.76	4.00
Bed length (cm)	119	237	8.8	1.0	0.95	2.0	1.9	122	0.975	126	7.1	1.0
Ratio bed diameter to GAC particle size	111	111	69	58	46	46	46	111	46	96	69	58
Hydraulic loading rate (m/h)	7.6	7.6	6.9	10.7	10.5	10.7	10.6	7.3	10.5	7.9	6.7	14.0
Bed density (g/cm^3)	0.54	0.54	0.54	0.54	0.54	0.54	0.54	0.5	0.5	0.41	0.41	0.41
Bed porosity	0.37	0.37	0.37	0.37	0.37	0.37	0.37	0.37	0.37	0.37	0.37	0.37
Re	5.9	5.9	0.38	0.62	0.55	0.55	0.55	5.88	0.55	7.0	0.37	0.78
Re-Sc	5403-9814	5403-9814	417-758	647-1175	639-1160	650-1180	645-1170	5167-9385	639-1162	6426-11672	407-739	859-1561
Biot number	93-111	93-111	33-37	38-44	38-44	38-44	38-44	91-109	38-44	100-120	32-37	42-49

[a] CD-RSSCT parameters are averages of two replicate RSSCTs, [b] RSSCT designed assuming proportionality factor of X=0.25, SS denotes stainless steel column; PP denotes polypropylene column

Table S5. Design specifications for RSSCTs conducted in Water A to evaluate effects of GAC particle size and design choice

Parameter	GAC 1			
	PD-RSSCT	PD-RSSCT	CD-RSSCT	CD-RSSCT
Water TOC (mg L^{-1})	1.3	1.3	1.5	1.5
Media US Standard mesh size	100x200	200x230	200x230	230x325
Log-mean GAC particle diameter (mm)	0.108	0.069	0.069	0.053
(Simulated) EBCT (min)	1.20 (10.2)	0.748 (10.0)	1.20 (10.1)	1.20 (10.0)
GAC mass (g)	1.29	0.846	0.0427	0.0258
Bed diameter (mm)	4.76	4.76	3.18	3.18
Bed length (cm)	13.5	8.8	1.0	0.6
Ratio bed diameter to GAC particle size	44	69	46	60
Hydraulic loading rate (m/h)	6.8	6.9	10.6	10.6
Bed density (g/cm^3)	0.54	0.54	0.54	0.54
Bed porosity	0.37	0.37	0.37	0.37
Re	0.59	0.38	0.55	0.43
Re-Sc	647-1175	429-779	650-1180	503-913
Biot number	38-44	33-38	38-44	35-40

Table S6. Design specifications for pilot-scale GAC adsorber and RSSCT for Water B

Parameter	GAC 1	
	Pilot	CD-RSSCT
Water TOC (mg L^{-1})	<0.5	<0.5
Media US Standard mesh size	12x40	200x230
Log-mean GAC particle diameter (mm)	0.92	0.069
Scaling factor	-	13.3
(Simulated) EBCT (min)	12.4	0.055 (9.8)
GAC mass (g)	2002	0.0427
Bed diameter (mm)	50.8	3.18
Bed length (cm)	182.9	0.95
Ratio bed diameter to GAC particle size	55	46
Hydraulic loading rate (m/h)	8.9	10.4
Bed density (g/cm^3)	0.54	0.54
Bed porosity	0.37	0.37
Re	6.9	0.54
Re-Sc	6312-11465	634-1151
Biot number	99-119	38-43

Table S7. Design specifications for pilot-scale GAC adsorbers and RSSCTs for Water C

Parameter	GAC 1		GAC 4		GAC 5		GAC 6	
	Pilot	CD-RSSCT	Pilot	CD-RSSCT	Pilot	CD-RSSCT	Pilot	CD-RSSCT
Water TOC (mg L^{-1})	1.3	1.6	1.3	1.6	1.3	1.6	1.3	1.6
Media US Standard mesh size	12x40	100x140	12x40	100x140	10x30	80x120	12x40	100x140
(Simulated) EBCT (min)	10.1	0.228 (12.0)[c]	10.1	0.228 (12.0)[c]	10.1 (10.0)	0.19 (11.3)[c]	10.1	0.228 (12.0)[c]
Log-mean GAC particle diameter (mm)	0.92	0.127	0.92	0.127	1.16	0.151	0.92	0.127
Scaling factor	-	7.2	-	7.2	-	7.7	-	7.2
GAC mass (g)	3344	0.55	3840	0.69	2353	0.34	3066	0.53
Bed diameter (mm)	77.2	7	77.2	7	77.2	7	77.2	7
Bed length (cm)	132	3.4	132	3.4	132	2.3	132	3.4
Ratio bed diameter to GAC particle size	84	55	84	55	67	46	84	55
Hydraulic loading rate (m/h)	7.88	7.95	7.88	7.95	7.88	6.70	7.88	7.95
Bed density (g/cm^3)	0.54	0.54	0.62	0.62	0.39	0.39	0.5	0.5
Bed porosity	0.37	0.37	0.37	0.37	0.37	0.37	0.37	0.37
Re	6.1	0.75	7.0	0.75	8.6	0.76	6.1	0.75
Re-Sc	7192-10175	1143-1617	7192-10175	1143-1617	9093-12865	1147-1622	7192-10175	1143-1617
Biot number	102-112	45-50	203-226	45-50	113-125	45-50	102-112	45-50

Table S8. Freundlich adsorption capacity and adsorption kinetic parameters for best fit of PSDM to PD- and CD-RSSCT data obtained with different GAC particle sizes, Carbon: GAC 1, EBCT: 10 minutes. Water A (TOC = 1.3 mgL^{-1}).

Compound	PD-RSSCT, 100x200 mesh			PD-RSSCT, 200x230 mesh			CD-RSSCT, 200x230 mesh			CD-RSSCT, 230x325 mesh		
	Freundlich K (L/g)	Tortuosity	SPDFR	Freundlich K (L/g)	Tortuosity	SPDFR	Freundlich K (L/g)	Tortuosity	SPDFR	Freundlich K (L/g)	Tortuosity	SPDFR
PFBA	24	1.0	1.0	23	1.0	1.0	10	2.0	1E-30	9	2.0	1E-30
PFPeA	39	1.0	1E-30	39	1.0	1E-30	16	2.0	1E-30	16	2.0	1E-30
PFHxA	68	2.0	1E-30	62	1.0	1E-30	32	3.0	1E-30	30	3.0	1E-30
PFHpA	120	3.0	1E-30	104	3.0	1E-30	37	3.0	1E-30	32	3.0	1E-30
PFOA	160	2.0	1E-30	170	3.0	1E-30	46	3.0	1E-30	42	3.0	1E-30
PFNA	210	2.0	1E-30	230	3.0	1E-30	56	3.0	1E-30	56	3.0	1E-30
PFDA	295	3.0	1E-30	305	3.0	1E-30	76	3.0	1E-30	76	3.0	1E-30
PFBS	85	3.0	1E-30	71	2.0	1E-30	30	2.0	1E-30	30	2.0	1E-30
PFHxS	180	2.0	1E-30	175	3.0	1E-30	57	3.0	1E-30	55	3.0	1E-30
PFOS	300	3.0	1E-30	340	4.0	1E-30	80	2.0	1E-30	70	2.0	1E-30
6:2 FTS	210	2.0	1E-30	210	3.0	1E-30	50	2.0	1E-30	45	2.0	1E-30
PFMOAA	22	1.0	1E-30	22	1.0	1E-30	12	2.0	1E-30	11	2.0	1E-30
PFO2HxA	55	1.0	1E-30	73	2.0	1E-30	38	3.0	1E-30	30	3.0	1E-30
PFO3OA	160	2.0	1E-30	165	2.0	1E-30	56	3.0	1E-30	52	3.0	1E-30
PFO4DA	280	3.0	1E-30	320	3.0	1E-30	88	3.0	1E-30	80	3.0	1E-30
PFO5DoA	--	--	--	--	--	--	130	3.0	1E-30	110	3.0	1E-30
PMPA	20	1.0	1E-30	25	1.0	1E-30	7	2.0	1E-30	6	2.0	1E-30
PEPA	24	1.0	1E-30	29	1.0	1E-30	12	2.0	1E-30	10	2.0	1E-30
GenX	35	1.0	1.0	41	1.0	1E-30	15	2.0	1E-30	14	2.0	1E-30
HydroEve	92	3.0	1E-30	95	3.0	1E-30	28	2.0	1E-30	26	2.0	1E-30
NVHOS	52	1.0	1E-30	62	1.0	1E-30	28	2.0	1E-30	26	2.0	1E-30
Nafion Byproduct 2	160	2.0	1E-30	155	3.0	1E-30	36	2.0	1E-30	35	2.0	1E-30

Table S9. Freundlich adsorption capacity and adsorption kinetic parameters for best fit of PSDM to pilot-scale and CD-RSSCT data, Water A (TOC =2.3 mgL^{-1}). Carbon: GAC 1, EBCT: 10 and 20 min.

Compound	GAC 1, simulated EBCT = 10 min						GAC 1, simulated EBCT = 20 min					
	Pilot-scale			CD-RSSCT			Pilot-scale			CD-RSSCT		
	Freundlich K (L/g)	Tortuosity	SPDFR	Freundlich K (L/g)	Tortuosity	SPDFR	Freundlich K (L/g)	Tortuosity	SPDFR	Freundlich K (L/g)	Tortuosity	SPDFR
PFBA	11	1.0	1.0	7	2.0	1E-30	12	1.0	1E-30	8	1.0	1E-30
PFPeA	13	2.0	1E-30	10	2.0	1E-30	16	1.0	1E-30	15	2.0	1E-30
PFHxA	16	2.0	1E-30	18	3.0	1E-30	19	1.0	1E-30	25	3.0	1E-30
PFHpA	22	2.0	1E-30	20	3.0	1E-30	25	1.0	1E-30	32	3.0	1E-30
PFOA	29	2.0	1E-30	25	3.0	1E-30	-	-	-	-	-	-
PFBS	-	-	-	-	-	-	-	-	-	-	-	-
PFHxS	36	1.0	1.0	30	3.0	1E-30	-	-	-	-	-	-
PFOS	-	-	-	-	-	-	-	-	-	-	-	-
PFMOAA	12	1.0	1E-30	8	2.0	1E-30	11.5	1.0	1.0	9	1.0	1E-30
PFO2HxA	18	1.0	1E-30	21	3.0	1E-30	19	1.0	1.0	28	3.0	1E-30
PFO3OA	32	2.0	1E-30	32	3.0	1E-30	33	1.0	1E-30	50	3.0	1E-30
GenX	8	1.0	1E-30	8	2.0	1E-30	15	1.0	1E-30	13	2.0	1E-30
Nafion Byproduct 2	32	1.0	1E-30	25	3.0	1E-30	-	-	-	-	-	-

Table S10. Freundlich adsorption capacity and adsorption kinetic parameters for best fit of PSDM to pilot-scale and CD-RSSCT data, Water A (TOC =2.3 mgL^{-1}). Carbon: GAC 2 and GAC 3, EBCT: 10 min.

Compound	GAC 2, simulated EBCT = 10 min						GAC 3, simulated EBCT = 10 min					
	Pilot-scale			CD-RSSCT			Pilot-scale			CD-RSSCT		
	Freundlich K (L/g)	Tortuosity	SPDFR	Freundlich K (L/g)	Tortuosity	SPDFR	Freundlich K (L/g)	Tortuosity	SPDFR	Freundlich K (L/g)	Tortuosity	SPDFR
PFBA	-	-	-	-	-	-	-	-	-	-	-	-
PFPeA	14	1.0	1.0	14	2.0	1E-30	-	-	-	-	-	-
PFHxA	18	1.0	1.0	22	2.0	1E-30	14	1.0	1E-30	15	2.0	1E-30
PFHpA	26	1.0	1E-30	26	2.0	1E-30	19	1.0	1E-30	21	2.0	1E-30
PFOA	35	1.0	1E-30	36	2.0	1E-30	24	1.0	1E-30	32	2.0	1E-30
PFBS	-	-	-	-	-	-	18	1.0	1E-30	18	2.0	1E-30
PFHxS	43	1.0	1.0	43	2.0	1E-30	33	2.0	1E-30	39	2.0	1E-30
PFOS	-	-	-	-	-	-	65	2.0	1E-30	70	2.0	1E-30
PFMOAA	11	1.0	1.0	9	1.0	1E-30	-	-	-	-	-	-
PFO2HxA	16	1.0	1.0	24	2.0	1E-30	-	-	-	-	-	-
PFO3OA	37	2.0	1E-30	40	2.0	1E-30	-	-	-	-	-	-
GenX	13	1.0	1.0	11	1.0	1E-30	11	1.0	1E-30	9	2.0	1E-30
Nafion Byproduct 2	38	1.0	1E-30	36	2.0	1E-30	-	-	-	-	-	-

Table S11: Freundlich adsorption capacity and adsorption kinetic parameters for best fit of PSDM to pilot-scale and PD-RSSCT data, Water A (TOC =2.3 mgL^{-1}). Carbon: GAC 1 and GAC 3, EBCT: 10 min.

Compound	GAC 1, simulated EBCT = 10 min						GAC 3, simulated EBCT = 10 min					
	Pilot-scale			PD-RSSCT			Pilot-scale			PD-RSSCT		
	Freundlich K (L/g)	Tortuosity	SPDFR	Freundlich K (L/g)	Tortuosity	SPDFR	Freundlich K (L/g)	Tortuosity	SPDFR	Freundlich K (L/g)	Tortuosity	SPDFR
PFBA	11	1.0	1.0	16	1.0	1E-30	-	-	-	-	-	-
PFPeA	13	2.0	1E-30	21	1.0	1E-30	-	-	-	-	-	-
PFHxA	16	2.0	1E-30	33	2.0	1E-30	14	1.0	1E-30	31	2.0	1E-30
PFHpA	22	2.0	1E-30	49	3.0	1E-30	19	1.0	1E-30	49	3.0	1.0
PFOA	29	2.0	1E-30	80	6.0	1E-30	24	1.0	1E-30	80	4.0	1E-30
PFBS	-	-	-	-	-	-	18	1.0	1E-30	36	2.0	1E-30
PFHxS	36	1.0	1.0	95	7.0	1E-30	33	2.0	1E-30	80	3.0	1E-30
PFOS	-	-	-	-	-	-	65	2.0	1E-30	225	7.0	1E-30
PFMOAA	12	1.0	1E-30	17	1.0	1.0	-	-	-	-	-	-
PFO2HxA	18	1.0	1E-30	41	3.0	1E-30	-	-	-	-	-	-
PFO3OA	32	2.0	1E-30	107	6.0	1E-30	-	-	-	-	-	-
GenX	8	1.0	1E-30	21	1.0	1E-30	11	1.0	1E-30	20	1.0	1E-30
Nafion Byproduct 2	32	1.0	1E-30	85	7.0	1E-30	-	-	-	-	-	-

Table S12. Freundlich adsorption capacity and adsorption kinetic parameters for best fit of PSDM to pilot-scale and CD-RSSCT data, Water B (TOC <0.5 mgL^{-1}). Carbon: GAC 1, EBCT: 10 min.

Compound	GAC 1, simulated EBCT = 10 min					
	Pilot-scale			CD-RSSCT		
	Freundlich K (L/g)	Tortuosity	SPDFR	Freundlich K (L/g)	Tortuosity	SPDFR
PFBA	44	1.0	3.0	30	1.0	1.0
PFPeA	65	1.0	5.0	63	1.0	1.0
PFHxA	80	1.0	3.0	85	1.0	0.5

Table S13. Freundlich adsorption capacity and adsorption kinetic parameters for best fit of PSDM to pilot-scale and RSSCT data obtained for PFOA in wastewater-impacted groundwater, Water C (TOC =1.3 mgL^{-1} and 1.6 mgL^{-1}, respectively). Carbon: GAC 1, 4, 5, and 6, EBCT: 10 min.

GAC	PFOA , simulated EBCT = 10 min					
	Pilot-scale			RSSCT		
	Freundlich K (L/g)	Tortuosity	SPDFR	Freundlich K (L/g)	Tortuosity	SPDFR
1	105	1.0	1E-30	112	2.0	1E-30
4	65	2.0	1E-30	70	3.0	1E-30
5	100	1.0	1E-30	80	2.0	1E-30
6	75	1.0	1E-30	100	1.0	1E-30

Table S14. Intraparticle diffusion scenarios for determining NTIF in RSSCTs.

Scenario	Tortuosity (τ)	Surface-pore diffusion flux ratio (SPDFR)	Normalized total intraparticle flux for RSSCT ($NTIF_{RSSCT}$)
Max pore diffusion	1	10^{-30}	1
Pore diffusion	>1	10^{-30}	1/τ
Pore plus surface diffusion	1	>10^{-30}	1+SPDFR

Table S15. Determination of adsorption kinetic parameters for different pilot-scale NTIF scenarios

Scenario	Scaled up normalized total intraparticle flux for pilot scale ($NTIF_{pilot\text{-}scale}$)	Tortuosity (τ)	Surface-pore diffusion flux ratio (SPDFR)
Mass pore diffusion	1	1	10^{-30}
Pore diffusion	<1	1/ $NTIF_{pilot\text{-}scale}$	10^{-30}
Pore plus surface diffusion	>1	1	$NTIF_{pilot\text{-}scale}$ - 1

Table S16. PSDM inputs resulting in the best fit to PFAS breakthrough curves obtained at the pilot-scale as well as PSDM inputs for scaling up CD-RSSCT data with proportionality factors (X) of 0, 0.25, 0.5, and 1.0. Carbon: GAC 1, EBCT: 10 minutes, Water A (TOC =2.3 mgL^{-1}).

	Pilot-scale			X=0.0			X=0.25			X=0.5			X=1.0		
Compound	Freundlich K (L/g)	Tortuosity	SPDFR	Freundlich K (L/g)	Tortuosity	SPDFR	Freundlich K (L/g)	Tortuosity	SPDFR	Freundlich K (L/g)	Tortuosity	SPDFR	Freundlich K (L/g)	Tortuosity	SPDFR
PFBA	11	1.0	1.0	7	2.0	1E-30	7	1.0	1E-30	7	1.0	0.8	7	1.0	5.7
PFPeA	13	2.0	1E-30	10	2.0	1E-30	10	1.0	1E-30	10	1.0	0.8	10	1.0	5.7
PFHxA	16	2.0	1E-30	18	3.0	1E-30	18	1.6	1E-30	18	1.0	0.2	18	1.0	3.5
PFHpA	22	2.0	1E-30	20	3.0	1E-30	20	1.6	1E-30	20	1.0	0.2	20	1.0	3.5
PFOA	29	2.0	1E-30	25	3.0	1E-30	25	1.6	1E-30	25	1.0	0.2	25	1.0	3.5
PFHxS	36	1.0	1.0	30	3.0	1E-30	30	1.6	1E-30	30	1.0	0.2	30	1.0	3.5
PFMOAA	12	1.0	1E-30	8	2.0	1E-30	8	1.0	1E-30	8	1.0	0.8	8	1.0	5.7
PFO2HxA	18	1.0	1E-30	21	3.0	1E-30	21	1.6	1E-30	21	1.0	0.2	21	1.0	3.5
PFO3OA	32	2.0	1E-30	32	3.0	1E-30	32	1.6	1E-30	32	1.0	0.2	32	1.0	3.5
GenX	8	1.0	1E-30	8	2.0	1E-30	8	1.0	1E-30	8	1.0	0.8	8	1.0	5.7
Nafion Byproduct 2	32	1.0	1E-30	25	3.0	1E-30	25	1.6	1E-30	25	1.0	0.2	25	1.0	3.5

Table S17. PSDM inputs resulting in the best fit to PFAS breakthrough curves obtained at the pilot-scale as well as PSDM inputs for scaling up CD-RSSCT data with proportionality factors (X) of 0, 0.25, 0.5, and 1.0. Carbon: GAC 1, EBCT: 20 minutes, Water A (TOC =2.3 mgL^{-1}).

	Pilot-scale			X=0.0			X=0.25			X=0.5			X=1.0		
Compound	Freundlich K (L/g)	Tortuosity	SPDFR	Freundlich K (L/g)	Tortuosity	SPDFR	Freundlich K (L/g)	Tortuosity	SPDFR	Freundlich K (L/g)	Tortuosity	SPDFR	Freundlich K (L/g)	Tortuosity	SPDFR
PFBA	12	1.0	1E-30	8	1.0	1E-30	8	1.0	0.9	8	1.0	2.7	8	1.0	12.4
PFPeA	16	1.0	1E-30	15	2.0	1E-30	15	1.0	1E-30	15	1.0	0.8	15	1.0	5.7
PFHxA	19	1.0	1E-30	25	3.0	1E-30	25	1.6	1E-30	25	1.0	0.2	25	1.0	3.5
PFHpA	25	1.0	1E-30	32	3.0	1E-30	32	1.6	1E-30	32	1.0	0.2	32	1.0	3.5
PFMOAA	11.5	1.0	1.0	9	1.0	1E-30	9	1.0	0.9	9	1.0	2.7	9	1.0	12.4
PFO2HxA	19	1.0	1.0	28	3.0	1E-30	28	1.6	1E-30	28	1.0	0.2	28	1.0	3.5
PFO3OA	33	1.0	1E-30	50	3.0	1E-30	50	1.6	1E-30	50	1.0	0.2	50	1.0	3.5
GenX	15	1.0	1E-30	13	2.0	1E-30	13	1.0	1E-30	13	1.0	0.8	13	1.0	5.7

Table S18. PSDM inputs resulting in the best fit to PFAS breakthrough curves obtained at the pilot-scale as well as PSDM inputs for scaling up CD-RSSCT data with proportionality factors (X) of 0, 0.25, 0.5, and 1.0. Carbon: GAC 2, EBCT: 10 minutes, Water A (TOC =2.3 mgL^{-1}).

	Pilot-scale			PSDM Scaling X=0.0			PSDM Scaling X=0.25			PSDM Scaling X=0.5			PSDM Scaling X=1.0		
Compound	Freundlich K (L/g)	Tortuosity	SPDFR	Freundlich K (L/g)	Tortuosity	SPDFR	Freundlich K (L/g)	Tortuosity	SPDFR	Freundlich K (L/g)	Tortuosity	SPDFR	Freundlich K (L/g)	Tortuosity	SPDFR
PFPeA	14	1.0	1.0	14	2.0	1E-30	14	1.0	1E-30	1.0	0.8	1.0	14	1.0	5.7
PFHxA	18	1.0	1.0	22	2.0	1E-30	22	1.0	1E-30	1.0	0.8	1.0	22	1.0	5.7
PFHpA	26	1.0	1E-30	26	2.0	1E-30	26	1.0	1E-30	1.0	0.8	1.0	26	1.0	5.7
PFOA	35	1.0	1E-30	36	2.0	1E-30	36	1.0	1E-30	1.0	0.8	1.0	36	1.0	5.7
PFHxS	43	1.0	1.0	43	2.0	1E-30	43	1.0	1E-30	1.0	0.8	1.0	43	1.0	5.7
PFMOAA	11	1.0	1.0	9	1.0	1E-30	9	1.0	0.9	1.0	2.7	1.0	9	1.0	12.4
PFO2HxA	16	1.0	1.0	24	2.0	1E-30	24	1.0	1E-30	1.0	0.8	1.0	24	1.0	5.7
PFO3OA	37	2.0	1E-30	40	2.0	1E-30	40	1.0	1E-30	1.0	0.8	1.0	40	1.0	5.7
GenX	13	1.0	1.0	11	1.0	1E-30	11	1.0	0.9	1.0	2.7	1.0	11	1.0	12.4
Nafion Byproduct 2	38	1.0	1E-30	36	2.0	1E-30	36	1.0	1E-30	1.0	0.8	1.0	36	1.0	5.7

Table S19. PSDM inputs resulting in the best fit to PFAS breakthrough curves obtained at the pilot-scale as well as PSDM inputs for scaling up CD-RSSCT data with proportionality factors (X) of 0, 0.25, 0.5, and 1.0. Carbon: GAC 3, EBCT: 10 minutes, Water A (TOC =2.3 mgL^{-1}).

	Pilot-scale			PSDM Scaling X=0.0			PSDM Scaling X=0.25			PSDM Scaling X=0.5			PSDM Scaling X=1.0		
Compound	Freundlich K (L/g)	Tortuosity	SPDFR	Freundlich K (L/g)	Tortuosity	SPDFR	Freundlich K (L/g)	Tortuosity	SPDFR	Freundlich K (L/g)	Tortuosity	SPDFR	Freundlich K (L/g)	Tortuosity	SPDFR
PFHxA	14	1.0	1E-30	15	2.0	1E-30	15	1.0	1E-30	15	1	1.0	15	1	6.7
PFHpA	19	1.0	1E-30	21	2.0	1E-30	21	1.0	1E-30	21	1	1.0	21	1	6.7
PFOA	24	1.0	1E-30	32	2.0	1E-30	32	1.0	1E-30	32	1	1.0	32	1	6.7
PFBS	18	1.0	1E-30	18	2.0	1E-30	18	1.0	1E-30	18	1	1.0	18	1	6.7
PFHxS	33	2.0	1E-30	39	2.0	1E-30	39	1.0	1E-30	39	1	1.0	39	1	6.7
PFOS	65	2.0	1E-30	70	2.0	1E-30	70	1.0	1E-30	70	1	1.0	70	1	6.7
GenX	11	1.0	1E-30	9	2.0	1E-30	9	1.0	1E-30	9	1	1.0	9	1	6.7

Table S20. PSDM inputs resulting in the best fit to PFAS breakthrough curves obtained at the pilot-scale as well as PSDM inputs for scaling up CD-RSSCT data with proportionality factors (X) of 0, 0.25, 0.5, and 1.0. Carbon: GAC 1, EBCT: 10 minutes, Water B (TOC <0.5 mgL^{-1}).

Compound	Pilot-scale			PSDM Scaling X=0.0			PSDM Scaling X=0.25			PSDM Scaling X=0.5			PSDM Scaling X=1.0		
	Freundlich K (L/g)	Tortuosity	SPDFR	Freundlich K (L/g)	Tortuosity	SPDFR	Freundlich K (L/g)	Tortuosity	SPDFR	Freundlich K (L/g)	Tortuosity	SPDFR	Freundlich K (L/g)	Tortuosity	SPDFR
PFBA	44	1.0	3.0	30	1.0	1.0	30	1.0	2.8	30	1.0	6.3	30	1.0	25.7
PFPeA	65	1.0	5.0	63	1.0	1.0	63	1.0	2.8	63	1.0	6.3	63	1.0	25.7
PFHxA	80	1.0	3.0	85	1.0	0.5	85	1.0	1.9	85	1.0	4.5	85	1.0	19.0

Table S21. PSDM inputs resulting in the best fit to PFOA breakthrough curves obtained at the pilot-scale with GACs 1, 4, 5, and 6 as well as PSDM inputs for scaling up CD-RSSCT data with proportionality factors (X) of 0, 0.25, 0.5, and 1.0. EBCT: 10 minutes, Water C (TOC =1.3 mgL^{-1} and 1.6 mgL^{-1}, respectively).

GAC	Pilot-scale			PSDM Scaling X=0.0			PSDM Scaling X=0.25			PSDM Scaling X=0.5			PSDM Scaling X=1.0		
	Freundlich K (L/g)	Tortuosity	SPDFR	Freundlich K (L/g)	Tortuosity	SPDFR	Freundlich K (L/g)	Tortuosity	SPDFR	Freundlich K (L/g)	Tortuosity	SPDFR	Freundlich K (L/g)	Tortuosity	SPDFR
1	105	1.0	1E-30	112	2.0	1E-30	112	1.2	1E-30	112	1.0	0.3	112	1.0	2.6
4	65	2.0	1E-30	70	3.0	1E-30	75	1.8	1E-30	75	1.1	1E-30	75	1.0	1.4
5	100	1.0	1E-30	80	2.0	1E-30	80	1.2	1E-30	80	1.0	0.4	80	1.0	2.9
6	75	1.0	1E-30	100	2.0	1E-30	100	1.2	1E-30	100	1.0	0.3	100	1.0	2.6

Table S22. Number of PFAS and percentage of data points for scaled up CD-RSSCT that were within 30% of the BV10% value determined in the pilot study for proportionality factors (X) of 0, 0.25, 0.5, and 1.

	GAC 1, 10 min EBCT, TOC = 2.3 mg/L		GAC 1, 20 min EBCT, TOC = 2.3 mg/L		GAC 2, 10 min EBCT, TOC = 2.3 mg/L		GAC 3, 10 min EBCT, TOC = 2.3 mg/L		Overall	
X	Number of PFAS	% total	Number of PFAS	% total	Number of PFAS	% total	Number of PFAS	% total	Number of PFAS	% total
0.0	3	27%	5	63%	2	20%	5	71%	15	42%
0.25	7	64%	7	88%	9	90%	4	57%	27	75%
0.5	4	36%	4	50%	8	80%	1	14%	17	47%
1.0	4	36%	4	50%	4	40%	0	0%	12	33%

Table S23. Number of PFAS and percentage of data points for scaled up CD-RSSCT that were within 30% of the BV20% value determined in the pilot study for proportionality factors (X) of 0, 0.25, 0.5, and 1.

	GAC 1, 10 min EBCT, TOC = 2.3 mg/L		GAC 1, 20 min EBCT, TOC = 2.3 mg/L		GAC 2, 10 min EBCT, TOC = 2.3 mg/L		GAC 3, 10 min EBCT, TOC = 2.3 mg/L		Overall	
X	Number of PFAS	% total	Number of PFAS	% total	Number of PFAS	% total	Number of PFAS	% total	Number of PFAS	% total
0.0	6	55%	7	88%	7	70%	6	86%	26	72%
0.25	7	64%	7	88%	9	90%	4	57%	27	75%
0.5	7	64%	4	50%	8	80%	2	29%	21	58%
1.0	4	36%	4	50%	4	40%	1	14%	13	36%

Table S24. Number of PFAS and percentage of data points for scaled up CD-RSSCT that were within 30% of the BV50% value determined in the pilot study for proportionality factors (X) of 0, 0.25, 0.5, and 1.

X	GAC 1, 10 min EBCT, TOC = 2.3 mg/L		GAC 1, 20 min EBCT, TOC = 2.3 mg/L		GAC 2, 10 min EBCT, TOC = 2.3 mg/L		GAC 3, 10 min EBCT, TOC = 2.3 mg/L		Overall	
	Number of PFAS	% total	Number of PFAS	% total	Number of PFAS	% total	Number of PFAS	% total	Number of PFAS	% total
0.0	7	64%	5	63%	9	90%	7	100%	28	78%
0.25	9	82%	5	63%	9	90%	5	71%	28	78%
0.5	9	82%	4	50%	9	90%	4	57%	26	72%
1.0	9	82%	3	38%	9	90%	4	57%	25	69%

Table S25. Number of PFAS and percentage of data points for scaled up CD-RSSCT that were within 30% of the BV70% value determined in the pilot study for proportionality factors (X) of 0, 0.25, 0.5, and 1.

X	GAC 1, 10 min EBCT, TOC = 2.3 mg/L		GAC 1, 20 min EBCT, TOC = 2.3 mg/L		GAC 2, 10 min EBCT, TOC = 2.3 mg/L		GAC 3, 10 min EBCT, TOC = 2.3 mg/L		Overall	
	Number of PFAS	% total	Number of PFAS	% total	Number of PFAS	% total	Number of PFAS	% total	Number of PFAS	% total
0.0	9	82%	3	38%	9	90%	6	86%	27	75%
0.25	9	82%	3	38%	9	90%	6	86%	27	75%
0.5	9	82%	4	50%	9	90%	6	86%	28	78%
1.0	8	73%	5	63%	9	90%	7	100%	29	81%

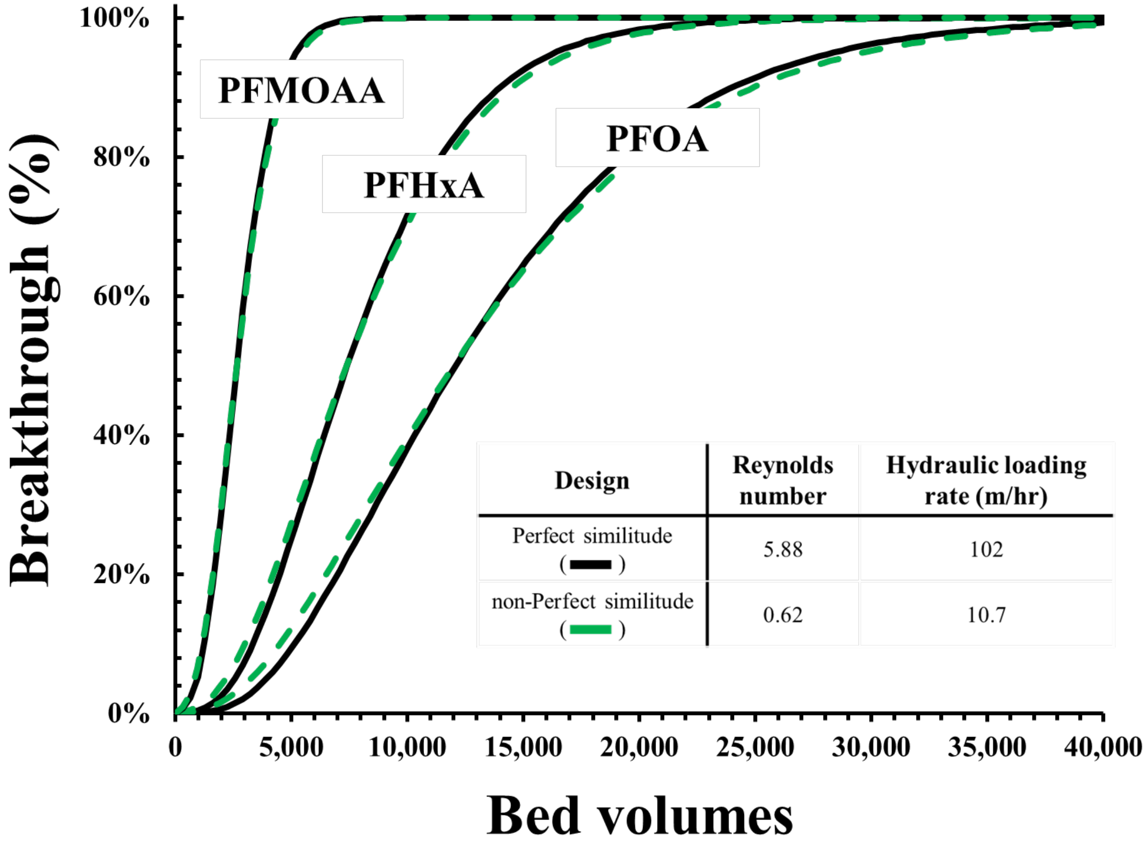

Figure S1. Comparison of simulated breakthrough curves using pore surface diffusion model for two constant diffusivity column designs.

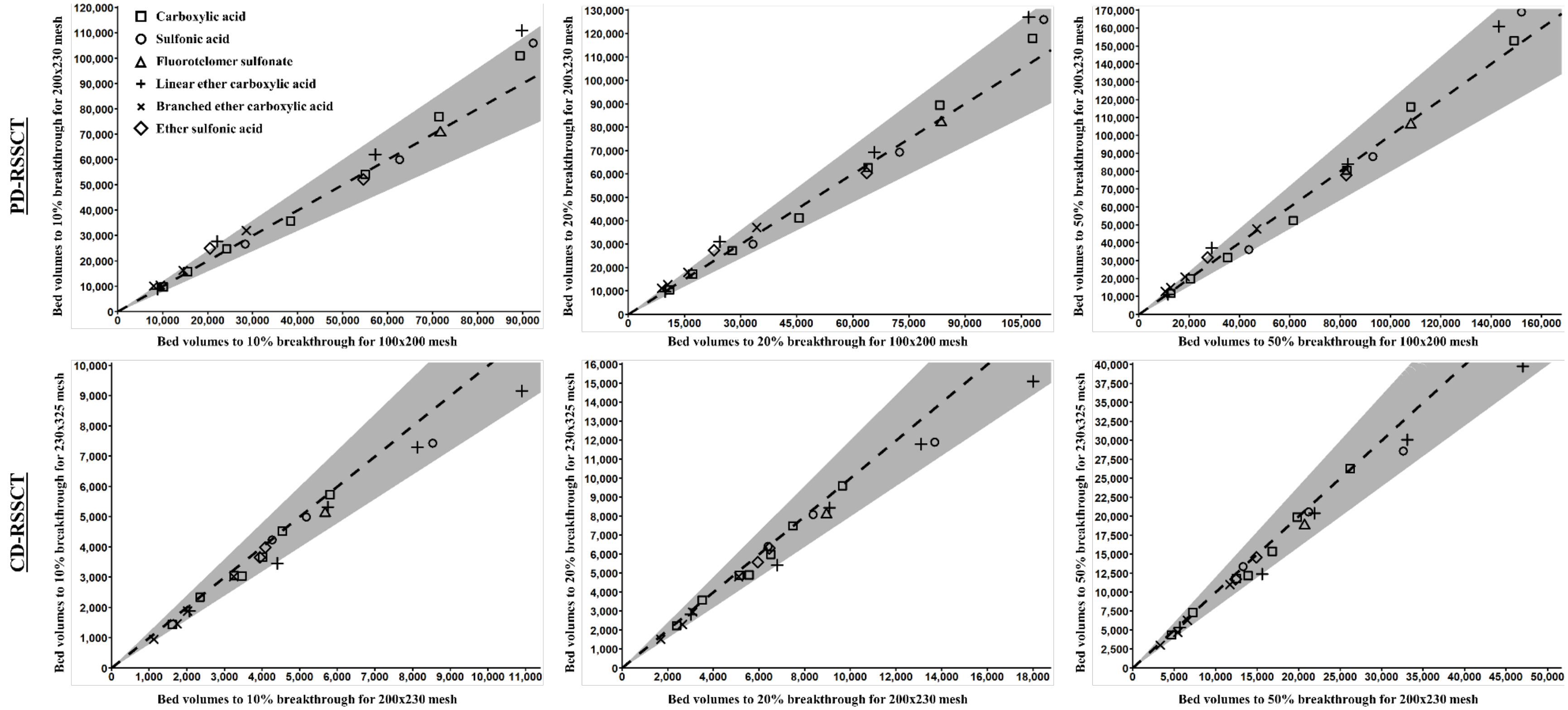

Figure S2. Comparison of bed volumes to 10, 20, and 50% breakthrough for PD-RSSCT at 100x200 and 200x230 mesh sizes in coagulated surface water (TOC = 1.3 mgL^{-1}) and for CD-RSSCT at 200x230 and 230x325 mesh sizes in coagulated surface water (TOC = 1.5 mgL^{-1}). Shaded regions indicate 20% deviation from perfect agreement (1:1 dashed line) between the two RSSCT designs. Carbon: GAC 1, Simulated EBCT: 10 minutes. Design specifications in Table S5.

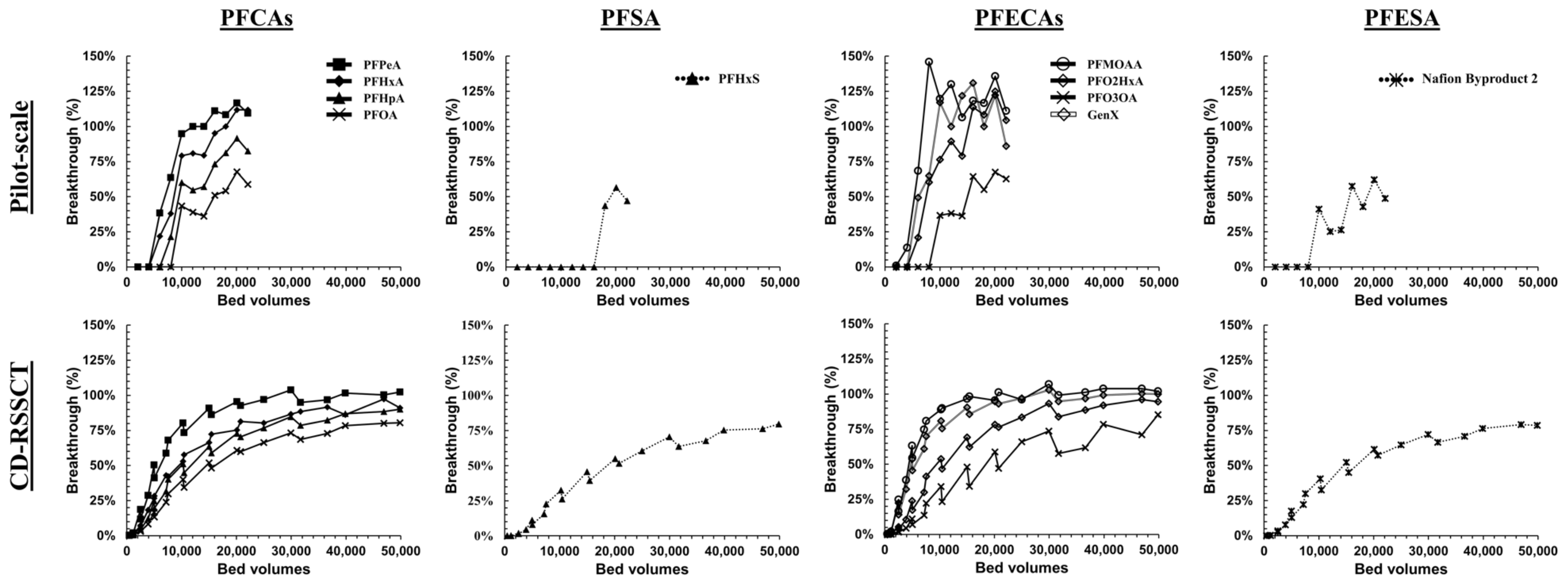

Figure S3. PFAS breakthrough curves obtained in Water A (TOC =2.3 mgL^{-1}) in a pilot-scale adsorber, CD-RSSCT, and PD-RSSCT. Carbon: GAC 2, Simulated EBCT: 10 minutes.

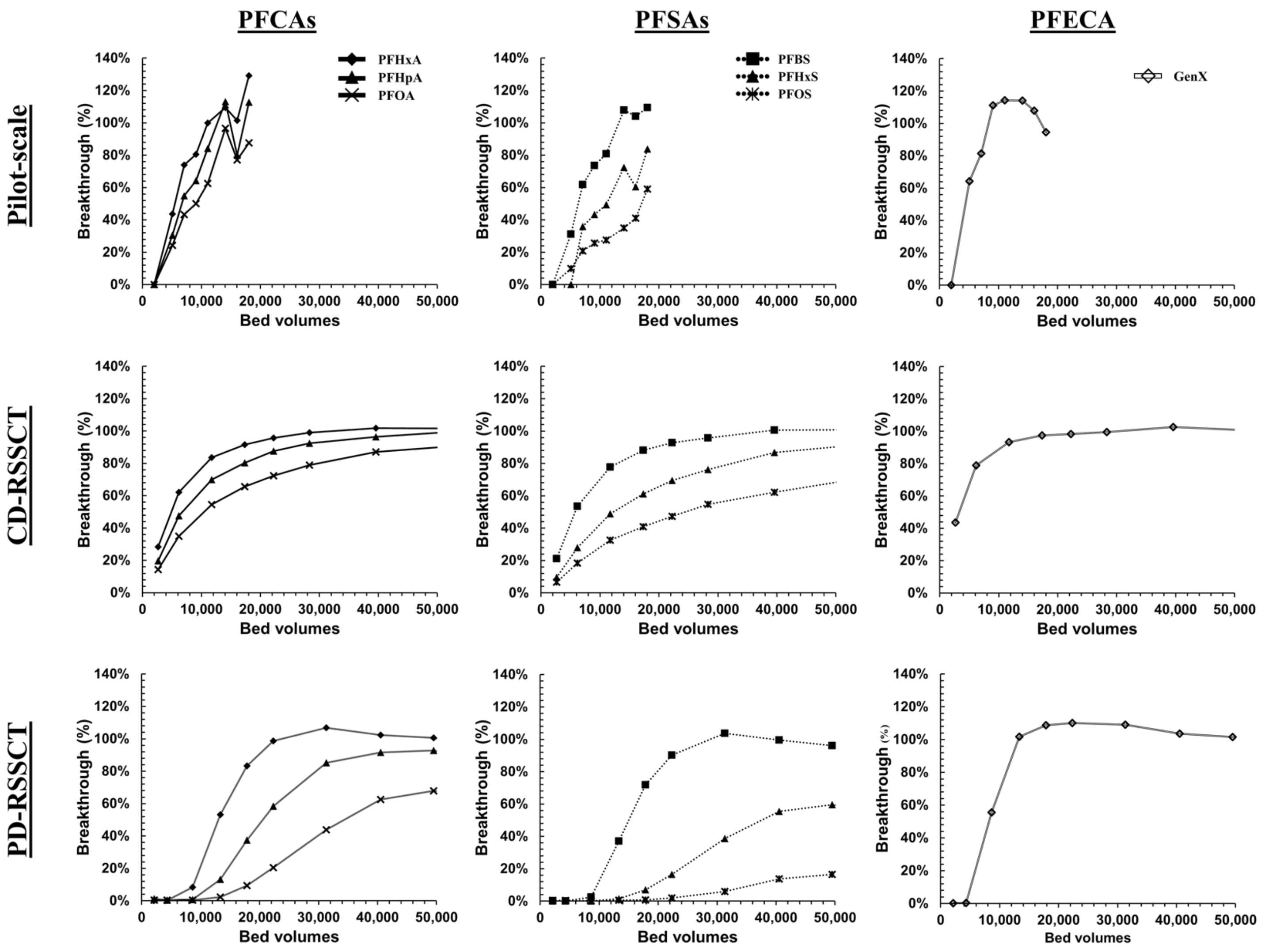

Figure S4. PFAS breakthrough curves obtained in Water A (TOC =2.3 mgL-1) in a pilot-scale adsorber, CD-RSSCT, and PD-RSSCT. Carbon: GAC 3, Simulated EBCT: 10 minutes.

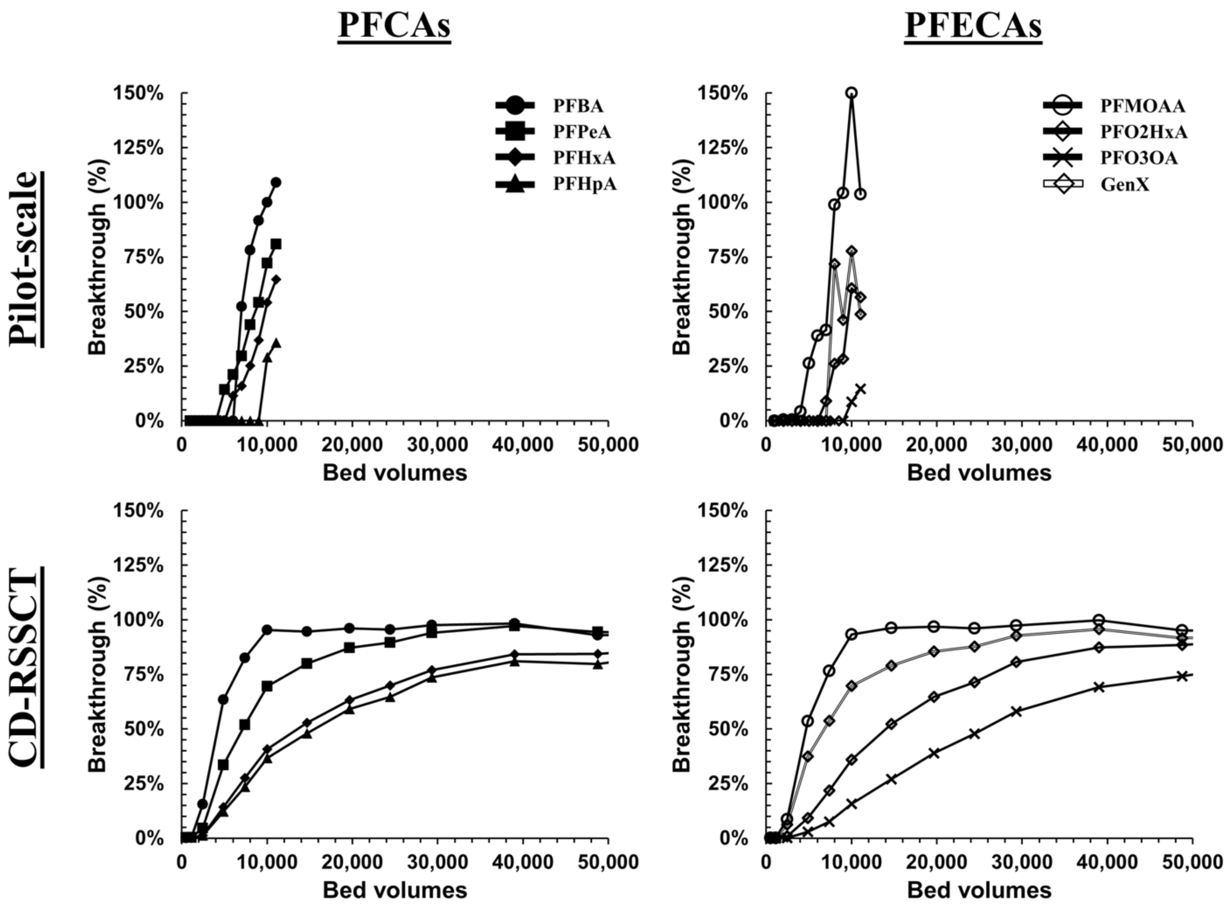

Figure S5. PFAS breakthrough curves obtained in Water A (TOC =2.3 mgL^{-1}) in a pilot-scale adsorber, CD-RSSCT, and PD-RSSCT. Carbon: GAC 1, Simulated EBCT: 20 minutes.

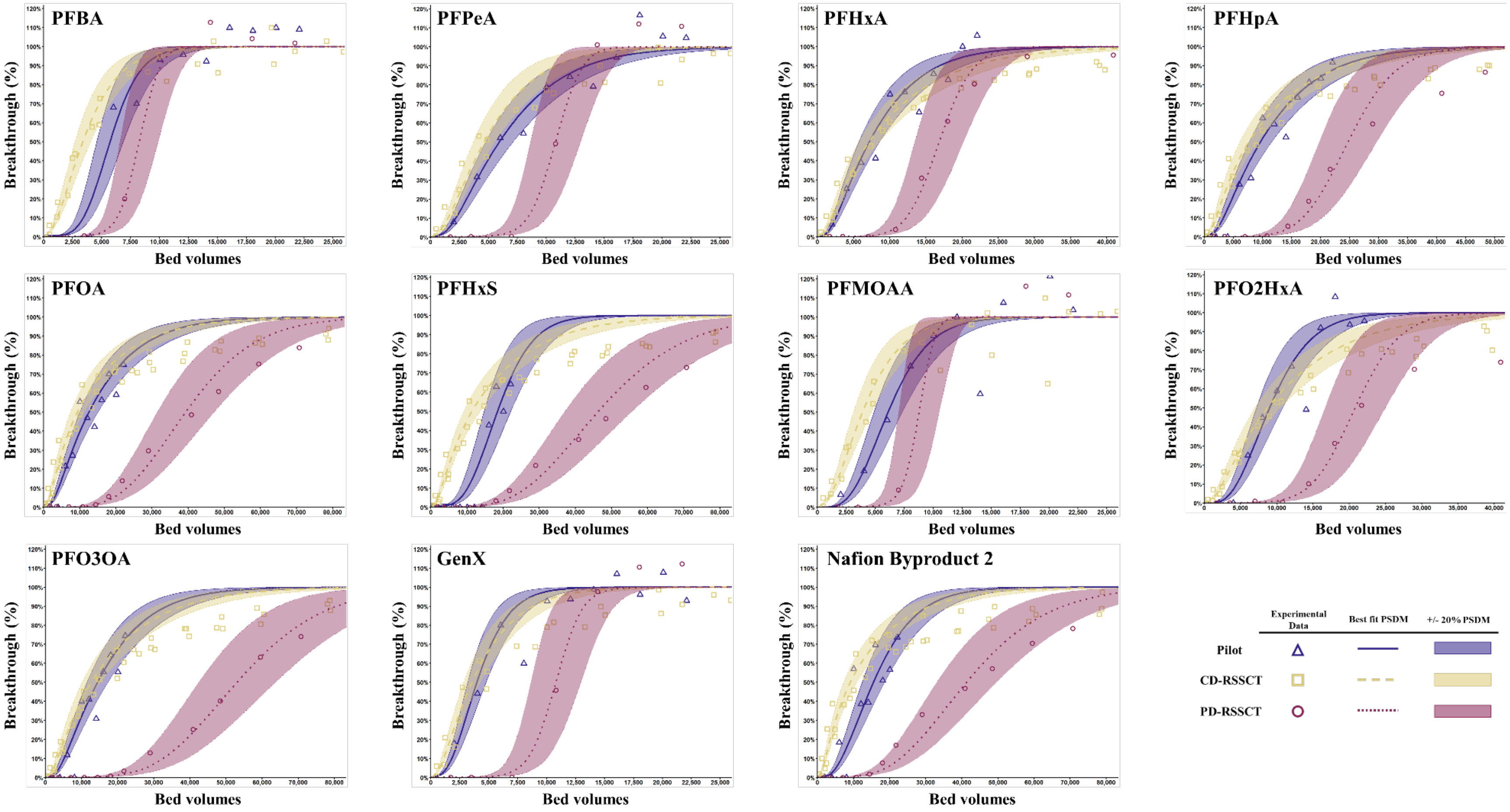

Figure S6. Direct comparison of PFAS breakthrough curves obtained at the pilot-scale (blue) and in RSSCTs: CD-RSSCT (red), PD-RSSCT (green). Water: coagulated/settled surface water (TOC = 2.3 mg L^{-1}), Carbon: GAC 1, simulated EBCT: 10 minutes. Symbols represent experimental data and lines represent best fit of the PSDM for each data set; the shaded region represents 20% variance around the best PSDM fit.

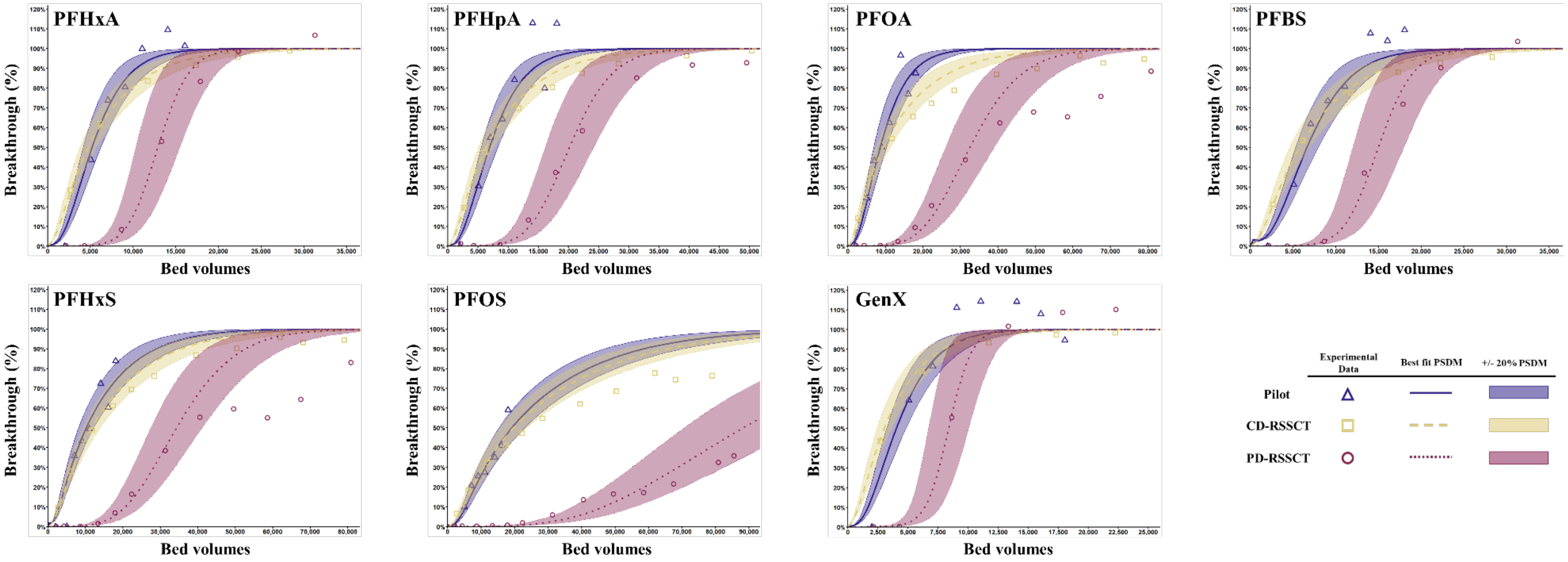

Figure S7. Direct comparison of PFAS breakthrough curves obtained at the pilot-scale (blue) and in RSSCTs: CD-RSSCT (red), PD-RSSCT (green). Water: coagulated/settled surface water (TOC = 2.3 mg L^{-1}), Carbon: GAC 3, simulated EBCT: 10 minutes. Symbols represent experimental data and lines represent best fit of the PSDM for each data set; the shaded region represents 20% variance around the best PSDM fit.

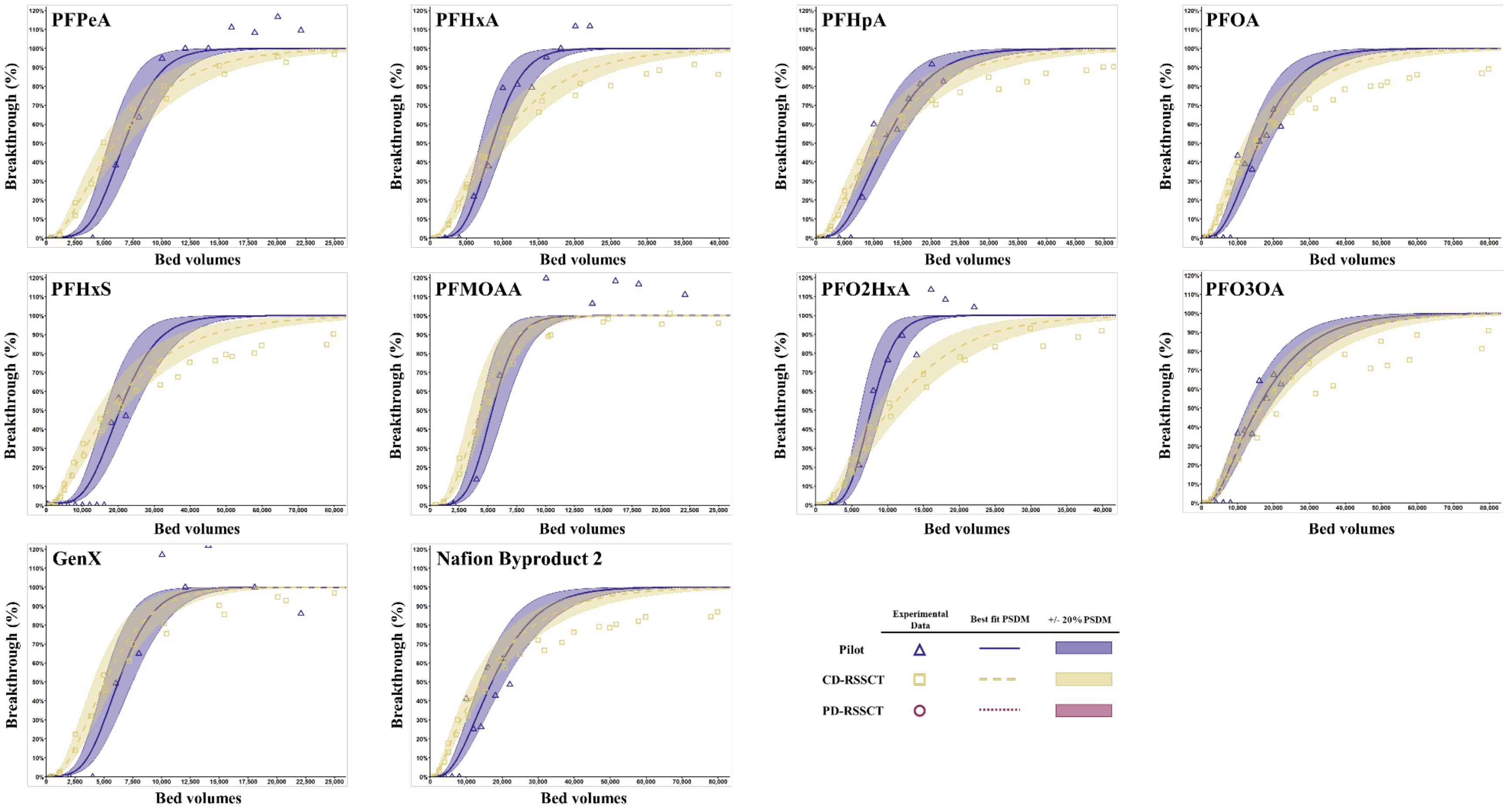

Figure S8. Direct comparison of PFAS breakthrough curves obtained at the pilot-scale (blue) and in a CD-RSSCT (red). Water: coagulated/settled surface water (TOC = 2.3 mg L^{-1}), Carbon: GAC 2, simulated EBCT: 10 minutes. Symbols represent experimental data and lines represent best fit of the PSDM for each data set; the shaded region represents 20% variance around the best PSDM fit.

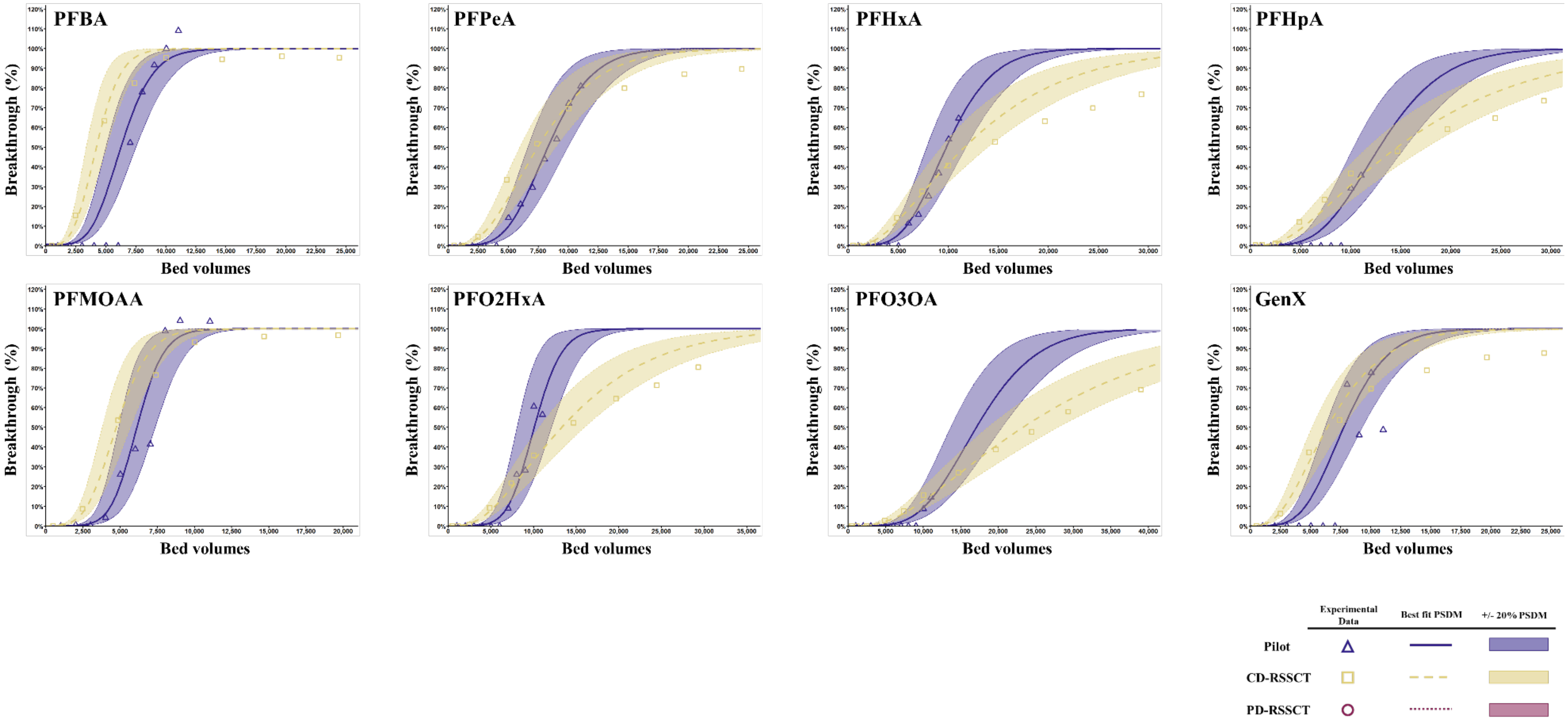

Figure S9. Direct comparison of PFAS breakthrough curves obtained at the pilot-scale (blue) and in a CD-RSSCT (red). Water: coagulated/settled surface water (TOC = 2.3 mg L^{-1}), Carbon: GAC 1, simulated EBCT: 20 minutes. Symbols represent experimental data and lines represent best fit of the PSDM for each data set; the shaded region represents 20% variance around the best PSDM fit.

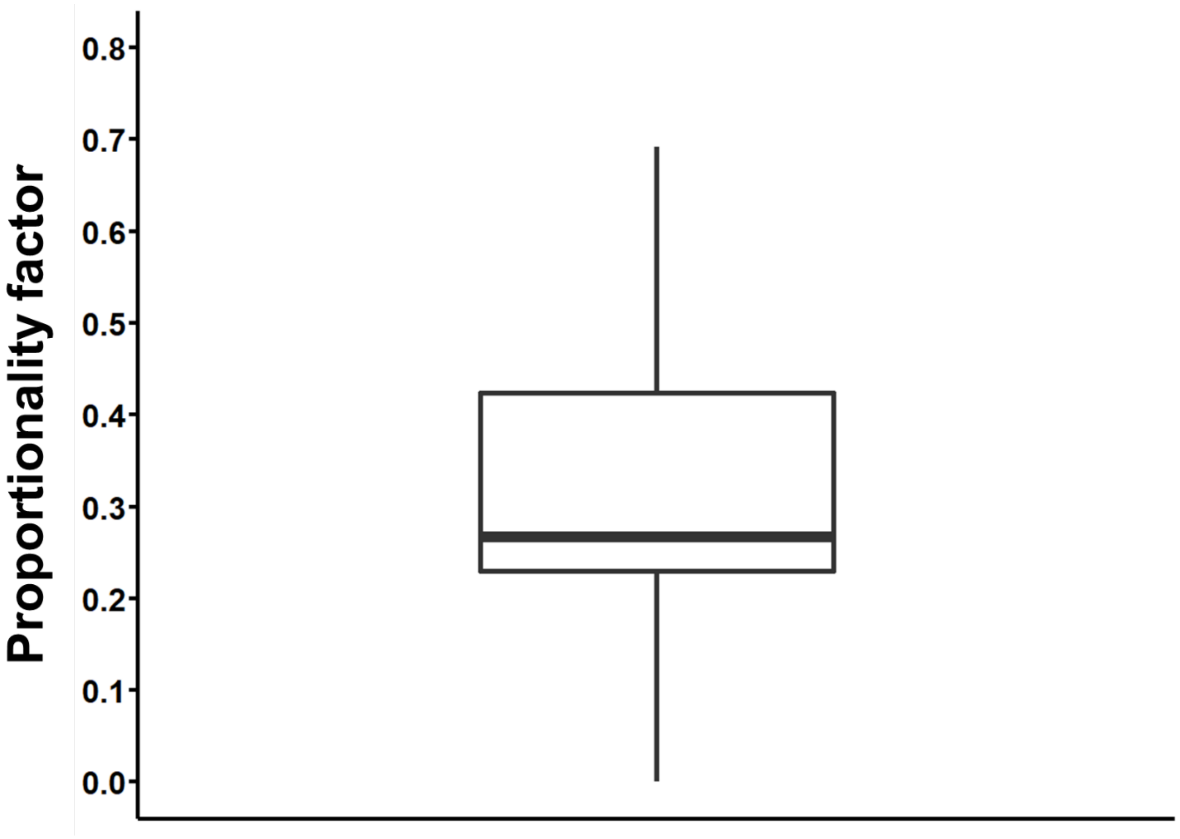

Figure S10. Median, interquartile range, and range of proportionality factors calculated from equation 3

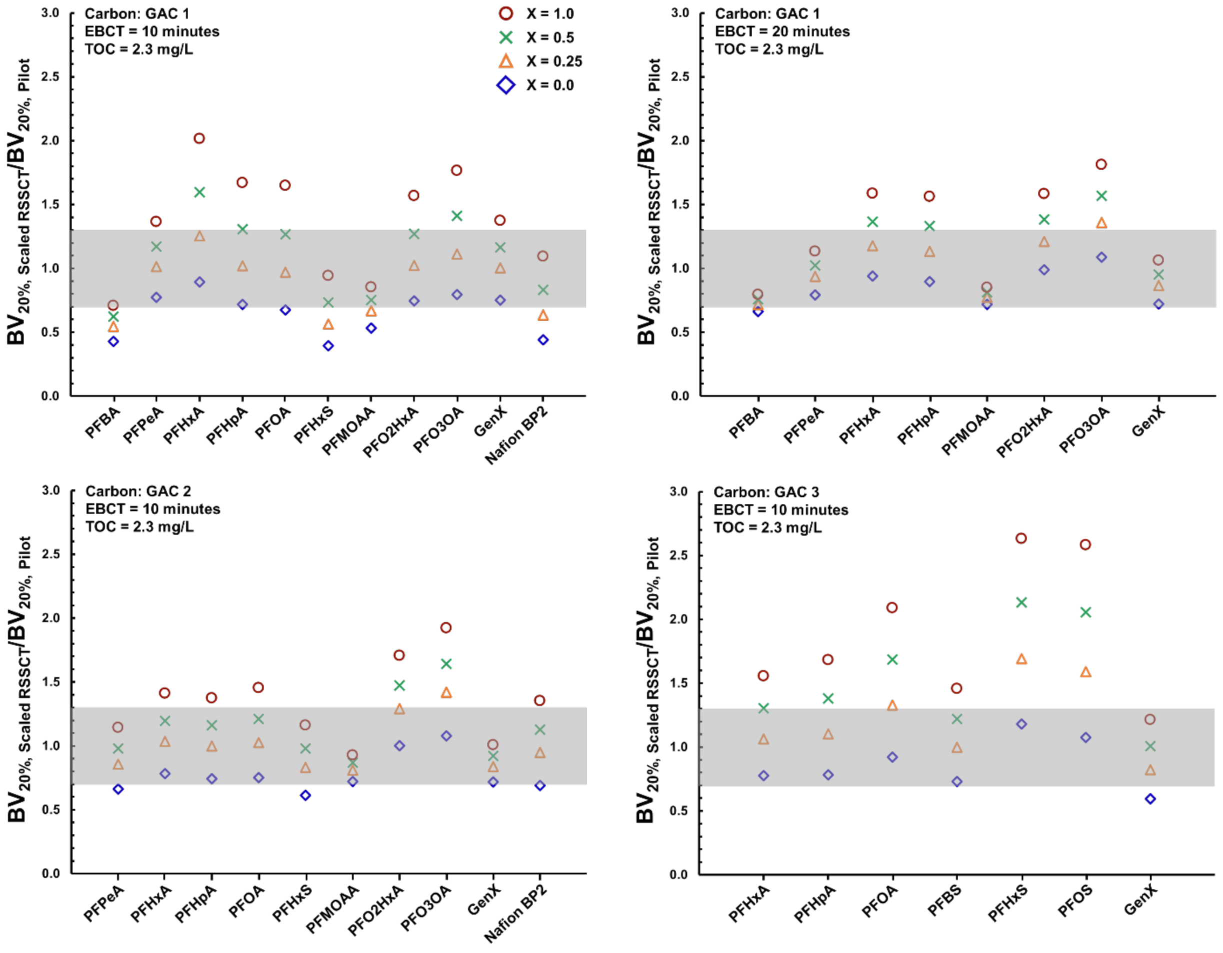

Figure S2. Comparison of bed volumes to 20% breakthrough for pilot-scale and scaled up CD-RSSCT data. Shaded region represents ±30% variance around perfect agreement.

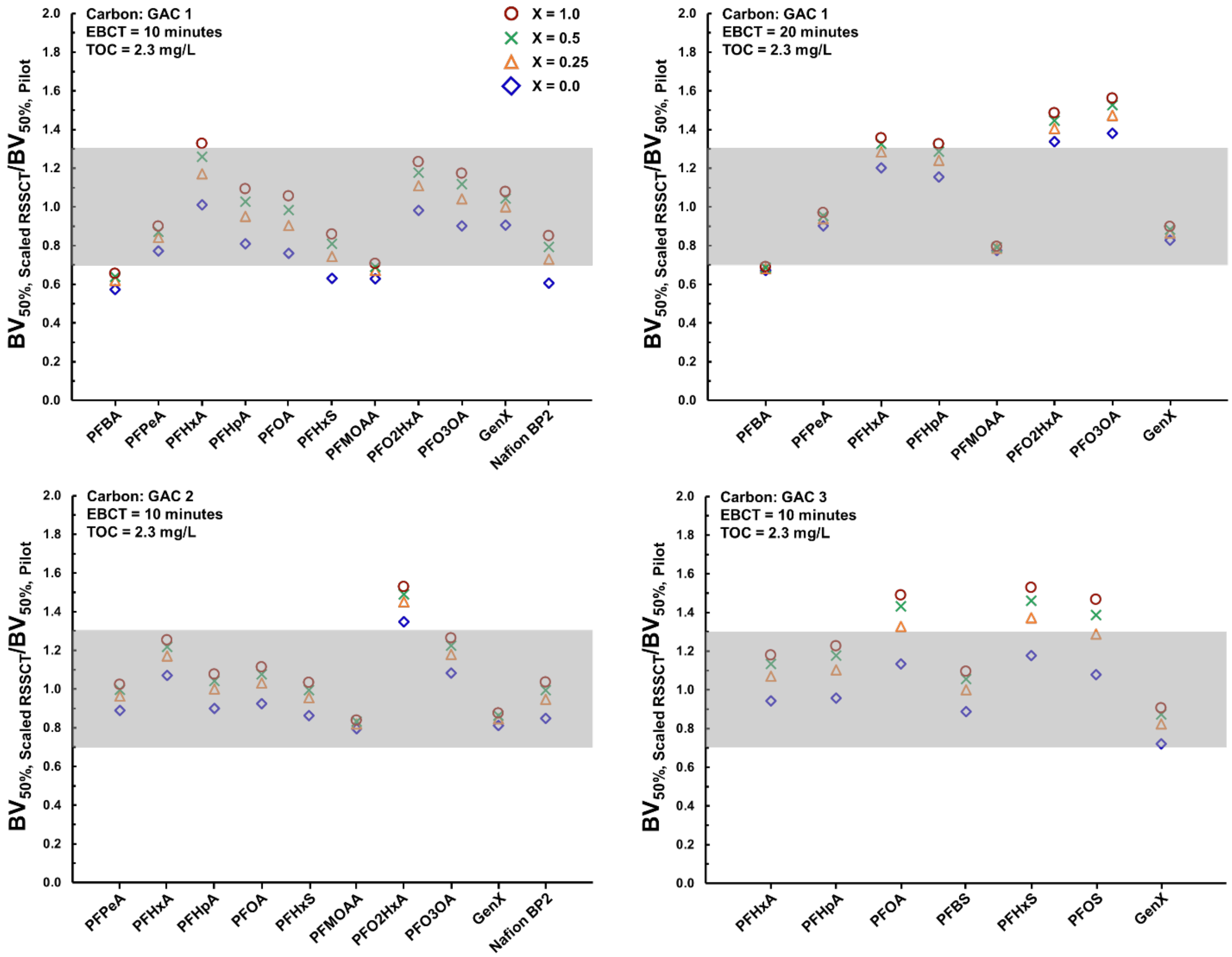

Figure S12. Comparison of bed volumes to 50% breakthrough for pilot-scale and scaled up CD-RSSCT data. Shaded region represents ±30% variance around perfect agreement.

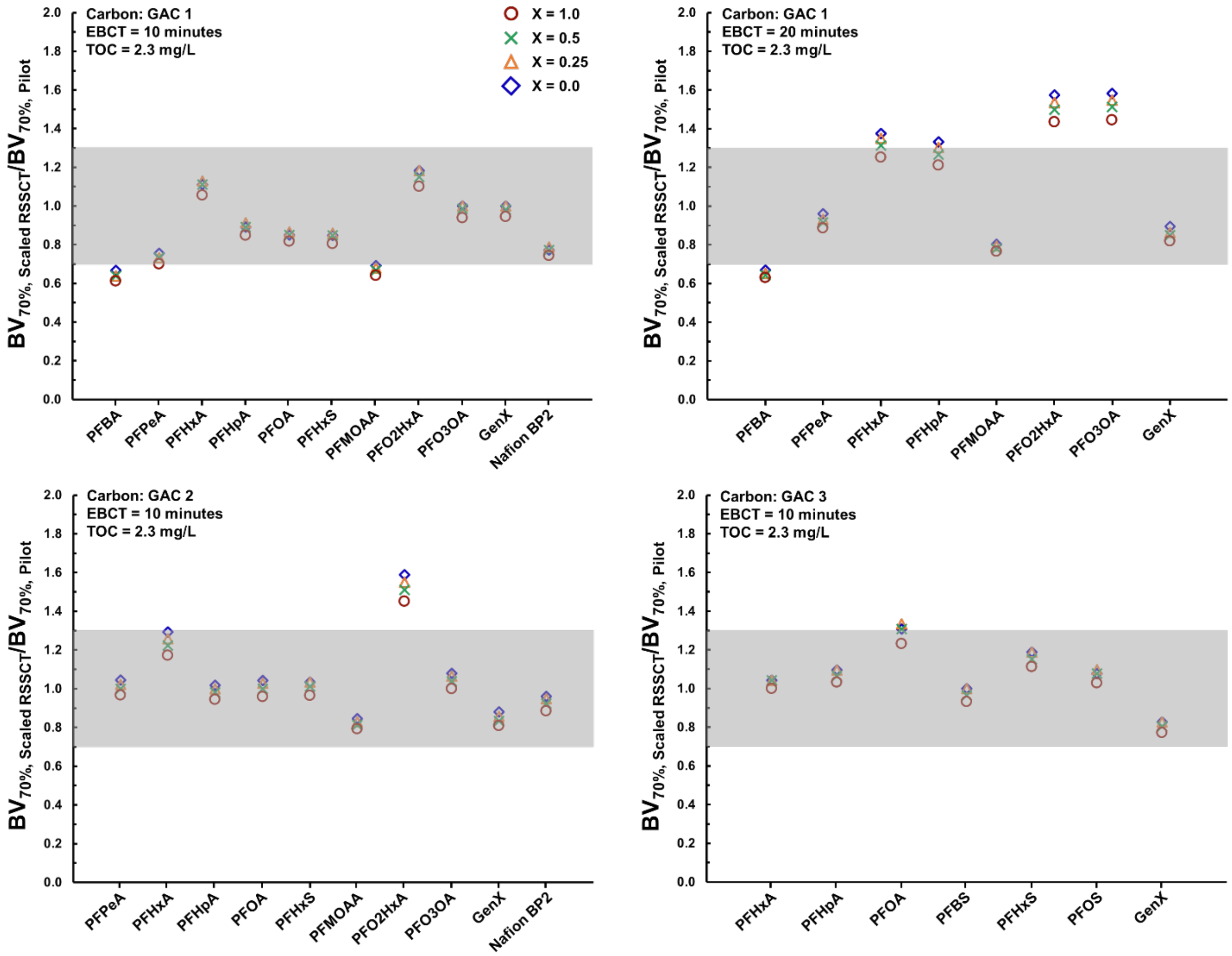

Figure S13. Comparison of bed volumes to 70% breakthrough for pilot-scale and scaled up CD-RSSCT data. Shaded region represents ±30% variance around perfect agreement.

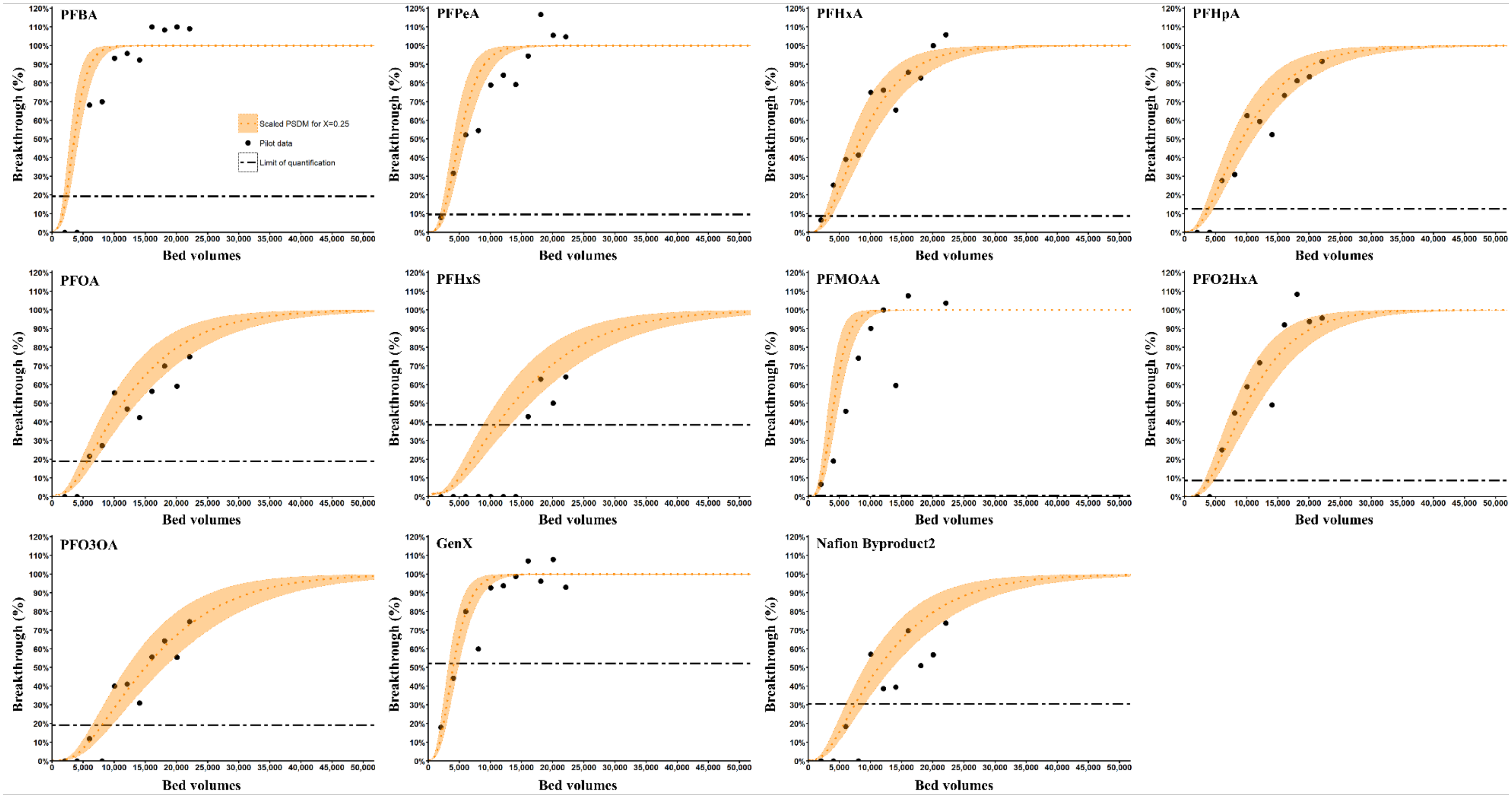

Figure S14. Comparison of PSDM predictions of pilot-scale PFAS breakthrough curves derived from CD-RSSCT data scaled up with a proportionality coefficient X of 0.25 and pilot-scale PFAS breakthrough data. Carbon: GAC 1, Water A (TOC: 2.3 mgL^{-1}), EBCT: 10 min. Shaded region represents +/-20% variance around the breakthrough curve prediction.

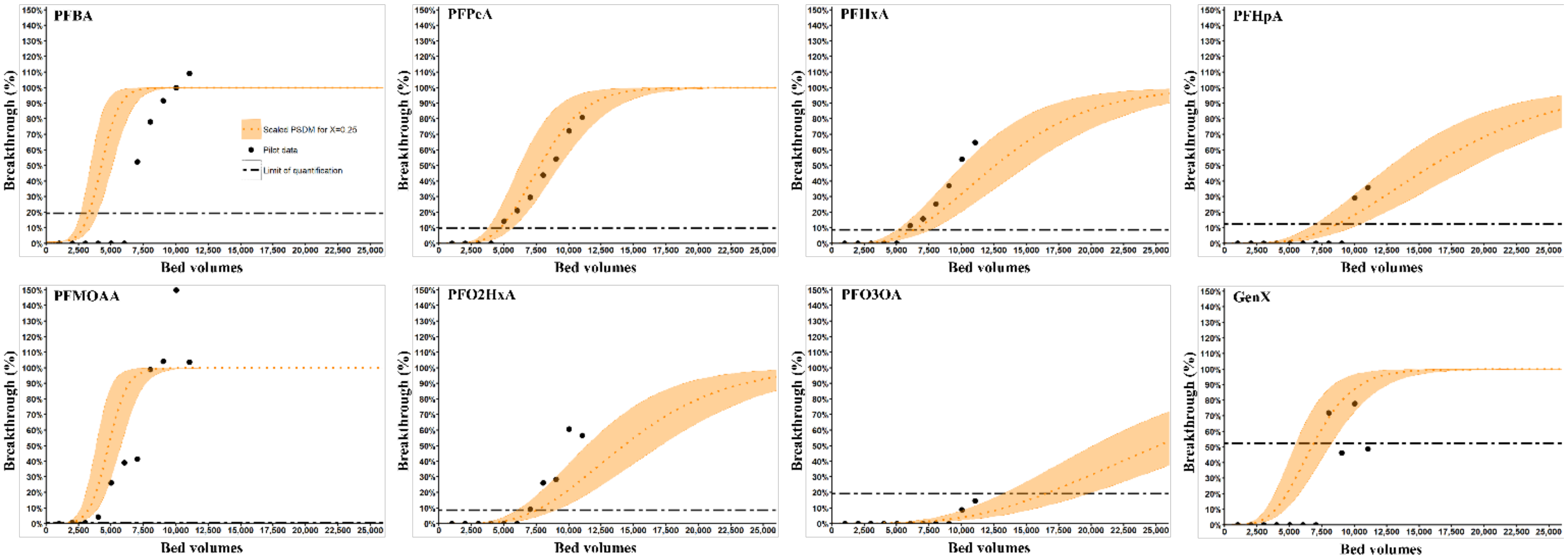

Figure S15. Comparison of pilot-scale data (black circles) and PSDM predictions derived from CD-RSSCT data scaled up with a proportionality coefficient X of 0.25 (dashed line). Carbon: GAC 1, Water A (TOC: 2.3 mgL^{-1}), EBCT: 20 min. Shaded region represents +/-20% variance around the breakthrough curve prediction. Horizontal dashed-dotted line represents the limit of quantification (LOQ) of the analytical method used to obtain the pilot-scale data.

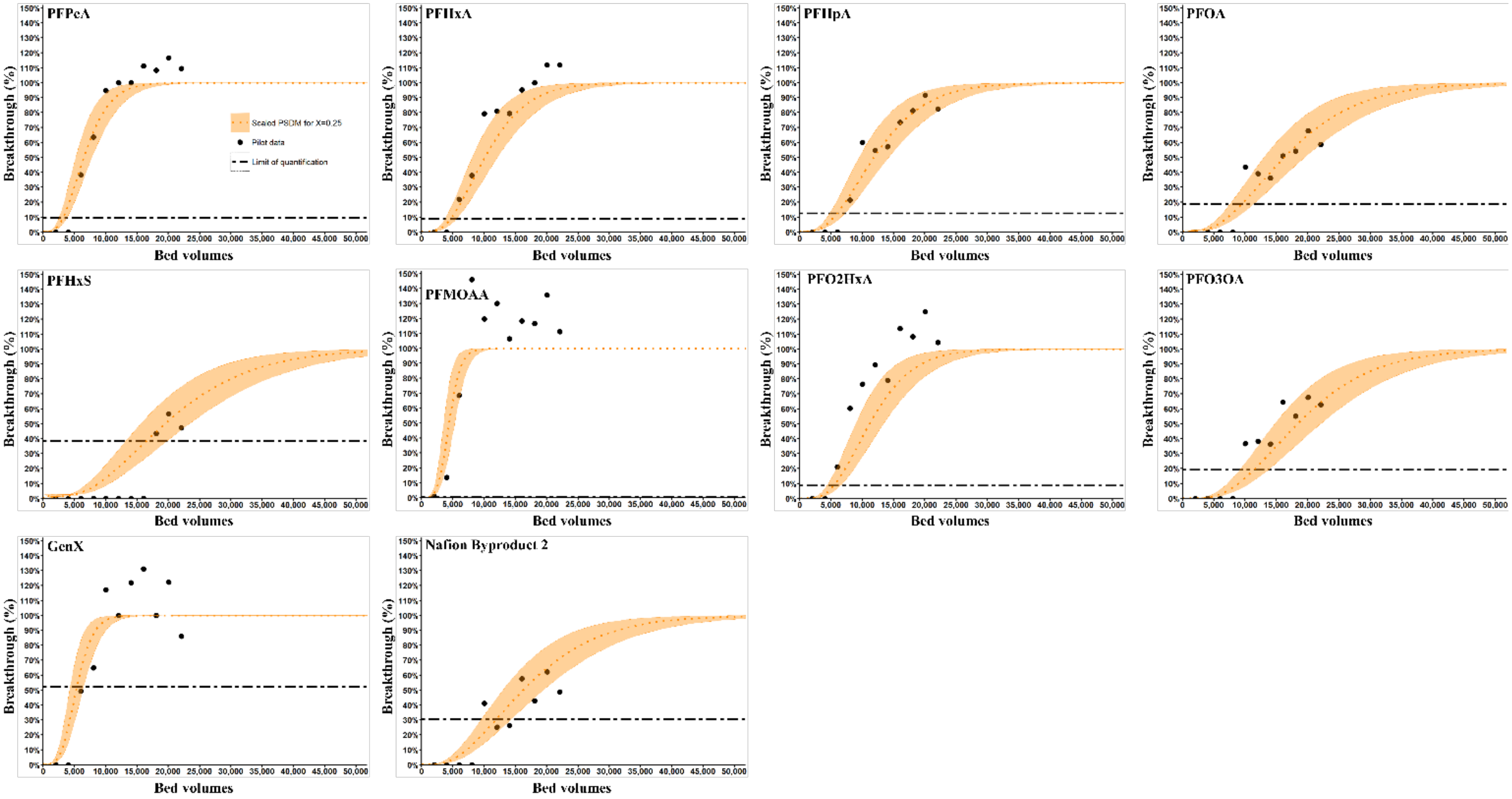

Figure S16. Comparison of pilot-scale data (black circles) and PSDM predictions derived from CD-RSSCT data scaled up with a proportionality coefficient X of 0.25 (dashed line). Carbon: GAC 2, Water A (TOC: 2.3 mgL^{-1}), EBCT: 10 min. Shaded region represents +/-20% variance around the breakthrough curve prediction. Horizontal dashed-dotted line represents the limit of quantification (LOQ) of the analytical method used to obtain the pilot-scale data.

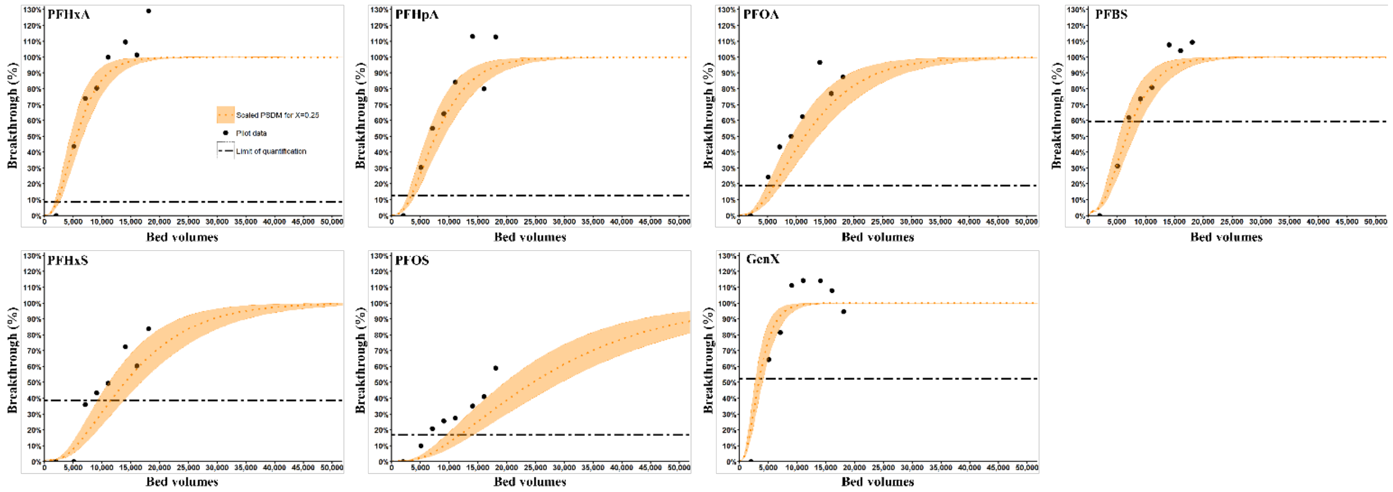

Figure S17. Comparison of pilot-scale data (black circles) and PSDM predictions derived from CD-RSSCT data scaled up with a proportionality coefficient X of 0.25 (dashed line). Carbon: GAC 3, Water A (TOC: 2.3 mgL^{-1}), EBCT: 10 min. Shaded region represents +/-20% variance around the breakthrough curve prediction. Horizontal dashed-dotted line represents the limit of quantification (LOQ) of the analytical method used to obtain the pilot-scale data.

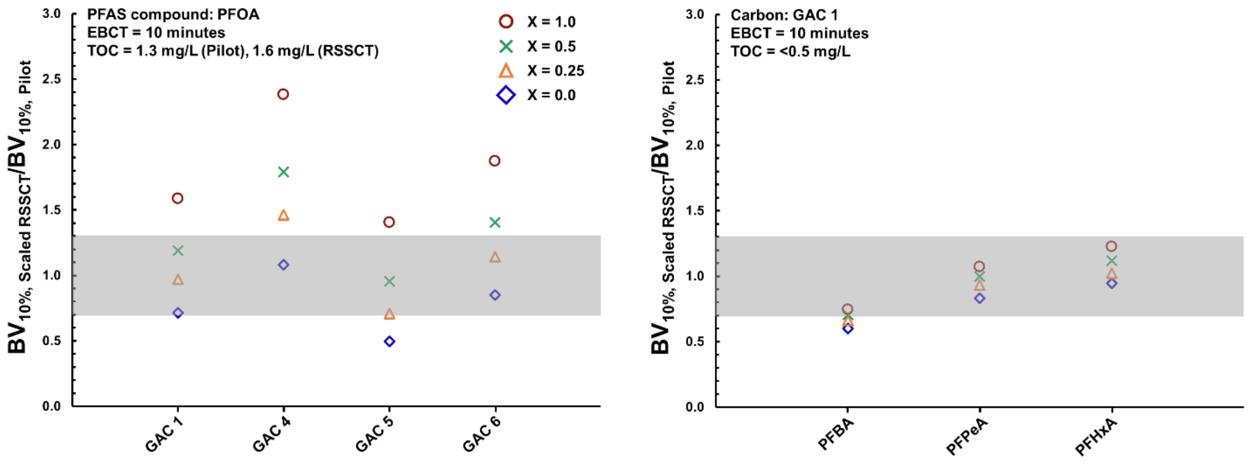

Figure S18. Comparison of bed volumes to 10% breakthrough for pilot-scale and scaled up CD-RSSCT data. Shaded region represents ±30% variance from perfect agreement. Left: PFOA data obtained with 4 GACs for Water C; Right) PFAS data obtained with GAC1 for Water B. EBCT: 10 min. Results are shown for CD-RSSCT data scaled up with proportionality factors of X=0.0 (blue diamond), X=0.25 (orange triangle) X=0.5 (green x), and X=1.0 (red circle).

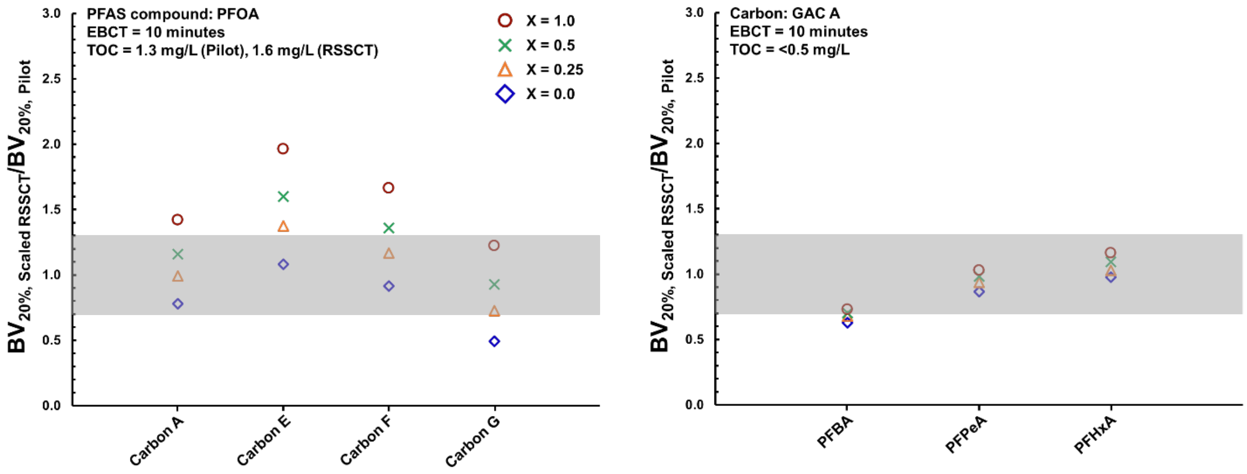

Figure S19. Comparison of bed volumes to 20% breakthrough for pilot-scale and scaled up CD-RSSCT data. Shaded region represents ±30% variance from perfect agreement. Left: PFOA data obtained with 4 GACs for Water C; Right) PFAS data obtained with GAC1 for Water B. EBCT: 10 min. Results are shown for CD-RSSCT data scaled up with proportionality factors of X=0.0 (blue diamond), X=0.25 (orange triangle) X=0.5 (green x), and X=1.0 (red circle).

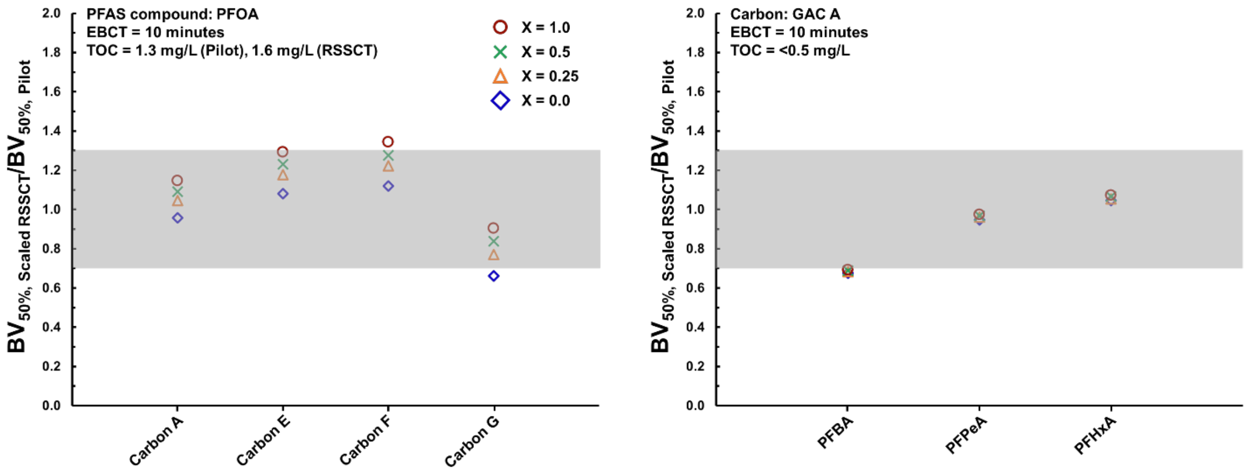

Figure S20. Comparison of bed volumes to 50% breakthrough for pilot-scale and scaled up CD-RSSCT data. Shaded region represents ±30% variance from perfect agreement. Left: PFOA data obtained with 4 GACs for Water C; Right) PFAS data obtained with GAC1 for Water B. EBCT: 10 min. Results are shown for CD-RSSCT data scaled up with proportionality factors of X=0.0 (blue diamond), X=0.25 (orange triangle) X=0.5 (green x), and X=1.0 (red circle).

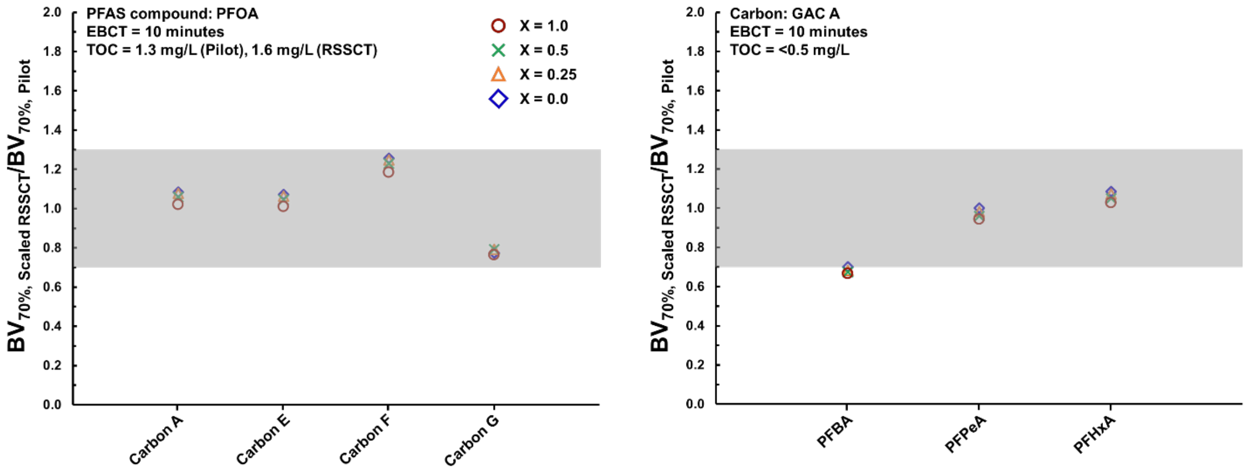

Figure S21. Comparison of bed volumes to 70% breakthrough for pilot-scale and scaled up CD-RSSCT data. Shaded region represents ±30% variance from perfect agreement. Left: PFOA data obtained with 4 GACs for Water C; Right) PFAS data obtained with GAC1 for Water B. EBCT: 10 min. Results are shown for CD-RSSCT data scaled up with proportionality factors of X=0.0 (blue diamond), X=0.25 (orange triangle) X=0.5 (green x), and X=1.0 (red circle).

References

Berrigan Jr, J.K. Scale-up of rapid small-scale adsorption tests to field-scale adsorbers: theoretical and experimental basis. M.S. Thesis, Michigan Technological University, 1985.

Chu, C.F.; & Ng, K.M. Flow in Packed Tubes with a Small Tube to Particle Diameter Ratio. AIChE Jour., 35.1 (1989):148.

Crittenden, J.C., Berrigan, J.K., Hand, D.W. "Design Of Rapid Small-Scale Adsorption Tests For A Constant Diffusivity." *Journal Water Pollution Control Federation* (1986): 312-319.

Crittenden, J.C., Berrigan, J.K., Hand, D.W., Lykins, B. "Design Of Rapid Fixed-Bed Adsorption Tests For Nonconstant Diffusivities." *Journal of Environmental Engineering* 113.2 (1987): 243-259.

Crittenden, J.C., P.S. Reddy, D.W. Hand, and H. Arora. *Prediction of GAC Performance Using Rapid Small-Scale Column Tests.* Denver, Colo.: Awwa Research Foundation, 1989.

Summers, R.S., Hooper, S.M., Solarik, G., Owen, D.M., and Hong. S. "Bench-Scale Evaluation of GAC for NOM Control." Journal AWWA 87.8 (1995): 69-80.

Summers, R. S., Kennedy, A.M., Knappe, D.R.U., Reinert, A.M., Fotta, E., Mastropole, A.J., Corwin, C.J., Roccaro, J. "Evaluation of Available Scale-Up Approaches for the Design of GAC Contactors." *Water Research Foundation*, Denver, CO, 2014.
